# *Trichinella spiralis* galectin binding to toll-like receptor 4 induces intestinal inflammation and mediates larval invasion of gut mucosa

**DOI:** 10.1186/s13567-023-01246-x

**Published:** 2023-11-27

**Authors:** Kai Ning Ma, Yao Zhang, Zhao Yu Zhang, Bo Ning Wang, Yan Yan Song, Lu Lu Han, Xin Zhuo Zhang, Shao Rong Long, Jing Cui, Zhong Quan Wang

**Affiliations:** Department of Parasitology, Medical College, Zhengzhou University, Zhengzhou, 450052 China

**Keywords:** *Trichinella spiralis*, galectin, Toll-like receptor 4 (TLR-4), gut epithelium, invasion, MAPK-NF-κB pathway

## Abstract

Previous studies showed that *Trichinella spiralis* galectin (Tsgal) facilitates larval invasion of intestinal epithelium cells (IECs). However, IEC proteins binding with Tsgal were not identified, and the mechanism by which Tsgal promotes larval invasion is not clear. Toll-like receptors (TLRs) are protein receptors responsible for recognition of pathogens. The aim of this study was to investigate whether recombinant Tsgal (rTsgal) binds to TLR-4, activates inflammatory pathway in gut epithelium and mediates *T. spiralis* invasion. Indirect immunofluorescence (IIF), GST pull-down and co-immunoprecipitation (Co-IP) assays confirmed specific binding between rTsgal and TLR-4 in Caco-2 cells. qPCR and Western blotting showed that binding of rTsgal with TLR-4 up-regulated the TLR-4 transcription and expression in Caco-2 cells, and activated p-NF-κB p65 and p-ERK1/2. Activation of inflammatory pathway TLR-4/MAPK-NF-κB by rTsgal up-regulated pro-inflammatory cytokines (IL-1β and IL-6) and down-regulated anti-inflammatory cytokine TGF-β in Caco-2 cells, and induced intestinal inflammation. TAK-242 (TLR-4 inhibitor) and PDTC (NF-κB inhibitor) significantly inhibited the activation of TLR-4 and MAPK-NF-κB pathway. Moreover, the two inhibitors also inhibited IL-1β and IL-6 expression, and increased TGF-β expression in Caco-2 cells. In *T. spiralis* infected mice, the two inhibitors also inhibited the activation of TLR-4/MAPK-NF-κB pathway, ameliorated intestinal inflammation, impeded larval invasion of gut mucosa and reduced intestinal adult burdens. The results showed that rTsgal binding to TLR-4 in gut epithelium activated MAPK-NF-κB signaling pathway, induced the expression of TLR-4 and pro-inflammatory cytokines, and mediated larval invasion. Tsgal might be regarded as a candidate molecular target of vaccine against *T. spiralis* enteral invasive stage.

**Supplementary Information:**

The online version contains supplementary material available at 10.1186/s13567-023-01246-x.

## Introduction

*Trichinella spiralis* is a worldwide zoonotic parasitic nematode of the genus *Trichinella*. *Trichinella* infection in humans results from the consumption of raw or poorly cooked animal meat containing infectious muscle larvae (ML) [1]. In China, eight outbreaks of human trichinellosis with 479 cases and 2 deaths were documented during 2009–2020, and 7 (87.50%) of the 8 outbreaks were involved in ingesting raw or semi-cooked pork [2]. *Trichinella* infection is a major foodborne zoonotic parasitic disease which is not only a significant public health problem but also a serious threat to meat food safety. However, it is hard to eradicate animal *Trichinella* infection as *Trichinella* has a variety of natural hosts and there are no available preventive vaccines [3, 4]. Hence, it is necessary to develop anti-*Trichinella* vaccines to intercept *Trichinella* infection in food animals and eliminate the infectious larvae in meat [5].

After the infected meat is ingested, *T. spiralis* ML are released from collagen capsules in skeletal muscles, develop to intestinal infectious larvae (IIL). The IIL penetrate intestinal epithelium cells (IEC), grow to adult worms (AW) after undergoing four molts, and the pregnant female adults give birth to the newborn larvae (NBL), which invade into skeletal muscles through lymphatic/bloodstream, and are encapsulated to accomplish the life cycle [6]. The IIL invasion of gut epithelia is the first key procedure for establishing *Trichinella* infection, but the mechanism of larval invasion of gut epithelia is not completely elucidated so far. Since gut mucosal epithelial cells are the first native physical barrier against worm penetration, and the main interaction sites between the IIL and host [7, 8], characterization of *Trichinella* invasive molecules and their receptors (or ligands) will be helpful to understand larval penetration mechanism, and be valuable to develop vaccines against *Trichinella* penetration and infection [9–11].

In our previous studies, a beta-galactoside-binding lectin of *T. spiralis* (Tsgal, GenBank: XM_003381608.1) was identified from the ML surface proteins by proteomics [12]. Tsgal belongs to tandem repeat (TR) type galectin and had two carbohydrate recognition domains (CRD) sequence motif binding to β-galactoside [13]. Subsequently, the Tsgal was cloned, expressed and identified, the results showed Tsgal was expressed at various *T. spiralis* developmental stages (ML, IIL, adults, and NBL), principally localized in the cuticle and stichosome of the nematode. Tsgal specifically bound to IECs and mediated larval penetration into IECs, whereas anti-rTsgal antibodies impeded the worm penetration [14]. Vaccination of mice with oral rTsgal vaccine elicited an intestinal mucosal sIgA response and systemic mixed Th1/Th2 response, and a significant immune protection against *T. spiralis* infection [15]. The results suggested that Tsgal interacted with IECs and participated in larval invasion of gut epithelium during early stage of *T. spiralis* infection. However, IEC proteins binding to Tsgal were not identified, and the mechanism of Tsgal action on larval invasion is not clear.

Toll-like receptors (TLRs) are important cell surface transmembrane molecules. As type I transmembrane proteins outside the cell, TLRs consist of structural domains rich in repetitive leucine sequences, while their intracellular structural domains are homologous to human interleukin-1 (IL-1) [16], and TLRs link with intra- and extracellular signaling pathway when being stimulated by binding pathogens such as parasites, leads to inflammatory and immune responses for maintaining homeostasis. In the process of nematode infection, various excretory/secretory products (ESP) and glycoproteins from the nematode activate TLRs-related immune or inflammatory responses [17]. Previous studies showed that TLR-4 expression in intestinal tissues of infected mice was significantly increased at 1–2 weeks after *T. spiralis* infection [18, 19], suggesting that TLR-4 might be involved in *T. spiralis* invasion of host gut mucosa and enteral pathogenesis.

When TLRs are exposed and in contact with pathogenic molecules, a series of signaling pathways such as NF-κB and MAPK pathways are triggered. The expression of TLR-4, MyD88 and NF-κB are up-regulated in host macrophages after *T. spiralis* infection, suggesting that *T. spiralis* infection regulated expression of TLR-4 on macrophages and TLR-4/MyD88/NF-κB pathways [20]. When TLRs are stimulated and the MAPK/NF-κB pathways are activated, cellular inflammatory response would be induced [21]. For example, Cathepsin B of *Naegleria fowleri* (NfCBs) was a pathogenic factor involved in the pathogenicity of amoeba; it elicited pro-inflammatory immune responses in BV-2 microglial cells through NF-κB and activator protein 1 (AP-1) dependent-MAPK pathway via TLR-4, induced an elevated secretion of several pro-inflammatory cytokines (TNF-α, IL-1α, IL-1β and IL-6), and exacerbated deleterious immune responses and tissue damages in *N. fowleri*-infected mouse brain [22]. The CRD of *Entamoeba histolytica* Gal/GalNAC lectin has been reported to bind to TLR-2 and TLR-4 in human colonic cells, and activated the NF-κB pathway and then enhanced adhesion of the trophozoites to the cells and cell damage [23]. *Haemonchus contortus* galectin was identified to have function in immune system by binding to glycoproteins on peripheral blood mononuclear cells (PBMC), and the glycoproteins may play an important role in cell migration, phagocytosis, and cytokine secretion [24]. However, it is not clear whether Tsgal binds to TLRs in host gut epithelium and participates in *T. spiralis* invasion of gut mucosa.

The aim of this study was to investigate whether rTsgal binds to TLR-4 receptor, activates MAPK/NF-κB pathway in IECs and mediates *T. spiralis* larval invasion of gut epithelium. The specific binding between Tsgal and TLR-4 was first identified by GST pull-down and Co-immunoprecipitation (Co-IP) technique, the downstream inflammatory signaling pathway associated with TLR-4 was investigated by qPCR and Western blotting. The specific inhibitors of TLR-4 receptor and pathway were also used the in vitro and in vivo experiment to further validate the roles of TLR-4 and MAPK/NF-κB pathway during intestinal *T. spiralis* infection. This study will help understand further the mechanisms of interaction between *T. spiralis* and the host gut epithelium, and provides new insights into Tsgal-mediated larval invasion in the process of *T. spiralis* infection.

## Materials and methods

### Parasite, experimental animal and cells

*Trichinella spiralis* isolate (ISS534) was collected from a naturally infected pig in Henan province of China, and passaged in BALB/c mice. Female mice, with 4-6-week-old were purchased from the Experimental Animal Center of Zhengzhou University. Human colonic epithelial cell line Caco-2 cell used in this study was obtained from the Cell Resource Center of the Shanghai Institute for Biological Sciences of the Chinese Academy of Sciences. All animal feeding and experimental procedures were approved by the Life Science Ethics Committee of Zhengzhou University (No. ZZUIRB GZR 2022-1317).

### Collection of *T. spiralis* IIL crude as well as ES antigens

Mice were orally infected with 10 000 *T. spiralis* ML. The infected mice were sacrificed at 6 h post-infection (hpi), the small intestine was collected and incubated in pre-warmed 37 °C saline for 2 h, then IIL were recovered as reported before [5, 12]. The collected IIL were washed with sterile PBS and cultured in RPMI-1640 (100 U penicillin/mL and 100 µg streptomycin/mL) at 37 °C for 18 h, the supernatant was collected, concentrated by low-speed ultrafiltration at 4000×*g* at 4 °C using an ultrafiltration tube to obtain IIL ES antigens [25]. The IIL crude antigens (CAg) were also prepared as described before [26].

### Cloning, expression and purification of GST-rTsgal

Tsgal gene was amplified by PCR from pQE-80L/Tsgal using specific primers with BamHI and EcoRI restriction sites (in **bold**) as follows: 5′-CGC**GGATCC**CCGTATT TAGCCAAGT-3′ and 5′-CCGCCG**GAATTC**TCATTCTAAATGAATCA-3′ [14]. To obtain recombinant Tsgal (rTsgal) with GST tag for the following GST pull-down assay, Tsgal gene was cloned into pGEX-4T-1 to construct recombinant expression plasmid pGEX-4T-1/Tsgal, rTsgal was expressed by induction with 1 mM IPTG at 16 °C for 3 d. The rTsgal with GST tag was purified with a GST-Sefinose (TM) Resin 4FF (Settled Resin) (Sangon Biotech., Shanghai, China), and was identified by SDS-PAGE with 12% acrylamide separating gel (80 V, 30 min and 120 V 90 min) [27, 28].

### Preparation of anti-rTsgal antibodies

Each of twenty BALB/c mice was immunized subcutaneously with 20 µg rTsgal emulsified with complete Freund’s adjuvant. Two boost immunizations were administered with 20 µg rTsgal emulsified with incomplete Freund’s adjuvant at a 2-week interval [29]. Two weeks after the third immunization, tail blood of immunized mice was collected to isolate anti-rTsgal immune serum samples. Serum anti-rTsgal IgG titer was measured by ELISA using rTsgal as coating antigen, as described previously [30].

### Western blot analysis

The IIL soluble crude antigens, ES antigens and rTsgal were separated by 12% separation gel on SDS-PAGE [15, 31]. The proteins were transferred to nitrocellulose (NC) membrane (Millipore, Billerica, USA) under the condition of 250 mA for 90 min [32] in the wet transfer cell (Bio-Rad, USA). After being blocked with 5% non-fat milk in Tris-buffered saline containing 0.05% Tween (TBST) at 37 °C for 2 h and washed with TBST, the membrane was cut into strips. The strips were probed by infection serum, anti-rTsgal serum and uninfected murine normal serum (1:100 dilutions in TBST) at 37 °C for 2 h. Following washes again, the strips were incubated at 37 °C for 1 h with HRP-conjugated anti-mouse IgG (1:10 000; Southern Biotech, Tuscaloosa, USA), and colored using 3,3′-diaminobenzidine tetrahydrochloride (DAB; Sigma-Aldrich, St. Louis, USA) or an enhanced chemiluminescent kit (AEC, Solarbio, Beijing, China) [28, 33].

Furthermore, expression level of TLR-4 in diverse groups of Caco-2 cells was also assessed in this study. Soluble proteins of Caco-2 cells treated with rTsgal, IIL CAg and lipopolysaccharide (LPS) were prepared and analyzed on SDS-PAGE and Western blotting [34, 35], and the TLR-4 expression level in Caco-2 cells was determined using Image J software. Each experiment had three replicates.

#### Caco-2 cell viability assessed by CCK-8 assay

Caco-2 cells were cultured in Dulbecco’s modified eagle medium (DMEM, Servicebio, Wuhan, China) supplemented with 10% fetal bovine serum (FBS, Gibco), 1% nonessential amino acid (NEAA, Sorlarbio, Beiing, China), 100 U/mL penicillin, and 0.1 mg/mL streptomycin. Caco-2 cells were cultured at 37 °C, 5% CO_2_ for 6 d in a 6-well plate until grown to the confluence, and the Caco-2 cells were digested [14, 27, 36]. Before rTsgal was used, the LPS in rTsgal was removed by High-Capacity Endotoxin Removal Spin Column (Thermo, USA), and the concentration of LPS in culture supernatant of recombinant *E. coli* was 0.05 ng/mL. CCK-8 kit (Meilunbio, Dalian, China) was used to detect the cell viability after being incubated with 0, 10, 20, 30, 40, 50 µg/mL rTsgal, or 0, 10, 20, 30, 40 µg/mL IIL crude antigens for 24 and 48 h [14], respectively. Then, CCK-8 solution (10 µL) was added to each well of 96-well plates, and incubated for 1 h. The absorbance at 450 nm was determined with a plate reader (Tecan, Schweiz, Switzerland).

#### Co-localization of rTsgal and TLR-4 in Caco-2 by indirect immunofluorescence (IIF)

Caco-2 cells were passaged on cover slip in a six-well plate until Caco-2 grown to confluence [10, 37], then incubated with rTsgal (50 µg/mL), IIL CAg (20 µg/mL, positive control) and PBS (negative control) at 37 °C for 4 h. The cells on the slip were immobilized using 4% formaldehyde and permeabilized using 0.25% Triton-X-100. The cells were blocked with 5% bovine serum albumin (BSA) in PBS for 1 h in 37 °C. Following washes with PBS three times, Caco-2 cells combined with rTsgal were probed by anti-rTsgal serum, infection serum and normal mouse serum (1:10 dilutions), TLR-4 in cell membranes were also probed by rabbit anti-TLR-4 antibody (1:100 dilutions; Abmart, Shanghai, China). Alexa Fluor 488-conjugated anti-mouse IgG (1:100; Abways, Shanghai, China) and the Cy3-conjugated anti-rabbit IgG (1:100; Abways) were used as the secondary antibody. 4′,6-Diamidino-2-phenylindole (DAPI) was used to dye the cell nucleus. Cellular localization of rTsgal and TLR-4 were observed by confocal microscopy (Olympus, Japan) [8].

### GST pull-down assay to verify the interaction between rTsgal and TLR-4

GST pull-down assay is based on the specific combination of glutathione mercaptotransferase (GST) with glutathione (GSH) in Sephrose 4B beads. The rTsgal fused with GST tag was purified, then interacted with its receptor TLR-4. The rTsgal binding with TLR-4 was captured, and the rTsgal-TLR-4 protein complex was denatured and identified by SDS-PAGE and Western blot analysis [38]. Briefly, the GST-rTsgal was first incubated with GSH conjugated resin (Sangon, Shanghai, China). After washes, the complex was incubated with Caco-2 cell soluble proteins. The GST tag in GSH conjugated resin was used as negative control. By Western blot analysis, antibodies against TLR-4 (1:1000 dilutions; Abmart), rTsgal and GST (1:100 dilutions) were used as the first antibodies, HRP-labeled goat anti-rabbit IgG and anti-mouse IgG (1:10 000 dilutions) were served as the second antibodies. After incubation (at 37 °C for 1 h) and washing, coloration was developed using an enhanced chemiluminescence kit (ECL, Solarbio) [9].

### Co-immunoprecipitation (Co-IP)

Co-IP and GST pull-down assay are mutually validated, and Co-IP is based on the specific binding of Protein A/G agarose system and IgG antibodies. To confirm whether rTsgal is able to bind to the receptor TLR-4 in Caco-2 cells, the Co-IP was performed as previously reported [39, 40]. In brief, 50 µg/mL rTsgal were first incubated with Caco-2 cells at 37 °C for 4 h, soluble cell proteins were prepared with DISC buffer (20 mM pH8.0 Tris-HCl, 1 mM EDTA, 200 mM NaCl). After centrifugation at 12 000 × *g* for 5 min, Protein A/G pre-conjugated with anti-rTsgal antibodies was added into the Caco-2 cell soluble protein supernatant, and incubated at 4 °C for 12 h, Caco-2 cell protein supernatant incubated with ProteinA/G-normal mouse IgG were used as a negative control. The beads were collected by centrifugation and washed three times with DISC lysis buffer; the bound proteins (protein A/G-rTsgal-TLR-4) were eluted and collected, and thermally denatured by boiling for 5 min. The protein samples were subjected to SDS-PAGE and transferred to a NC membrane. The membranes were blocked with 5% non-fat milk diluted in TBST at 37 °C for 2 h and cut into strips. The strips were probed by anti-TLR-4 antibody (1:1000 dilutions; Abmart) and anti-rTsgal serum (1:100 dilutions) at 37 °C for 1 h. After washing, the strips were incubated with HRP-conjugated goat anti-rabbit IgG or HRP-conjugated goat anti-mouse IgG (1:10 000; Southern Biotech, USA) at 37 °C for 1 h [26].

### qPCR

Total RNAs from Caco-2 cells and intestinal tissues were extracted using TRIzol reagent (Invitrogen, Carlsbad, USA) and reverse-transcribed into cDNA using PrimeScript RT reagent Kit (TaKaRa, Japan). qPCR was performed to assess the transcription level of various target genes in various groups of Caco-2 cells and intestinal tissues as described previously [41, 42]. The primer sequences for the tested genes are shown in Table 1. Human GAPDH (GenBank: NM_002046.7) and mouse GAPDH (GenBank: NM_008084) were used as internal control genes [43]. Relative mRNA expression levels of each tested gene were normalized by subtracting the mRNA expression level of an internal control gene, and the results were calculated with the 2^−ΔΔCt^ method as previously reported [37].

**Table 1:** **Specific primer sequences of Caco-2 and mouse makers and cytokines for qPCR**

Gene	Primers	Sequence	GenBank no.
GAPDH (Human)	Forward Reverse	5′-GTCTCCTCTGACTTCAACAGCG′-3′ 5′-ACCACCCTGTTGCTGTAGCCAA′-3′	NM_002046
TLR-4 (Human)	Forward Reverse	5′-AGACCTGTCCCTGAACCCTA′-3′ 5′-CTCCCAGAACCAAACGATG′-3′	NM_138554
NF-κB p65 (Human)	Forward Reverse	5′-TGAACCGAAACTCTGGCAGCTG′-3′ 5′-CATCAGCTTGCGAAAAGGAGCC′-3′	NM_021975
IL-1β (Human)	Forward Reverse	5′-CCACAGACCTTCCAGGAGAATG′-3′ 5′-GTGCAGTTCAGTGATCGTACAGG′-3′	NM_000576
IL-6 (Human)	Forward Reverse	5′-AGACAGCCACTCACCTCTTCAG′-3′ 5′-TTCTGCCAGTGCCTCTTTGCTG′-3′	NM_000600
TGF-β (Human)	Forward Reverse	5′-TACCTGAACCCGTGTTGCTCTC′-3′ 5′-GTTGCTGAGGTATCGCCAGGAA′-3′	NM_000660
GAPDH (Mouse)	Forward Reverse	5′-CATCACTGCCACCCAGAAGACTG′-3′ 5′-ATGCCAGTGAGCTTCCCGTTCAG′-3′	NM_008084
TLR-4 (Mouse)	Forward Reverse	5′-AGCTTCTCCAATTTTTCAGAACTTC′-3′ 5′-TGAGAGGTGGTGTAAGCCATGC′-3′	NM_021297
NF-κB p65 (Mouse)	Forward Reverse	5′-TCCTGTTCGAGTCTCCATGCAG′-3′ 5′-GGTCTCATAGGTCCTTTTGCGC′-3′	NM_009045
IL-1β (Mouse)	Forward Reverse	5′-TGGACCTTCCAGGATGAGGACA′-3′ 5′-GTTCATCTCGGAGCCTGTAGTG′-3′	NM_008361
IL-6 (Mouse)	Forward Reverse	5′-TACCACTTCACAAGTCGGAGGC′-3′ 5′-CTGCAAGTGCATCATCGTTGTTC′-3′	NM_031168
TGF-β (Mouse)	Forward Reverse	5′-TGATACGCCTGAGTGGCTGTCT′-3′ 5′-CACAAGAGCAGTGAGCGCTGAA′-3′	NM_011577

### Determination of MAPK/NF-κB pathway activation after rTsgal binding to TLR-4 in Caco-2

To investigate whether rTsgal binding with TLR-4 activates the MAPK-NF-κB pathway, relative transcription level of NF-κB p65 in Caco-2 cells treated with rTsgal, IIL CAg and LPS for 48 h was assessed by q-PCR [44]. To assess the activation of MAPK-NF-κB pathway, expression level of phosphorylated ERK1/2 and NF-κB p65 (p-ERK1/2 and p-NF-κB p65), and total ERK1/2 and NF-κB p65 were ascertained by Western blot. Caco-2 cells were first incubated with rTsgal, IIL CAg and LPS for 0.5 and 2 h, respectively. Soluble cell proteins were prepared in RIPA buffer containing protease inhibitors. The proteins were separated by SDS-PAGE and transferred on NC membrane; the membrane was blocked 5% BSA in TBST at 37 °C for 2 h. The primary antibodies against p-NF-κB p65 (1:1000 dilutions), NF-κB p65 (1:5000 dilutions), ERK1/2 (1:1000 dilutions) and p-ERK1/2 (1:1000 dilutions; Abmart) were used, HRP-conjugated anti-rabbit/mouse IgG (1:10 000 dilution) served as secondary antibodies.

### Determination of MAPK/NF-κB pathway by using inhibitors

TAK-242 (Resatovid) is a TLR-4 specific inhibitor which can selectively binds to the domains of TLR-4 in cells to block TLR-4 binding to signal molecules, so the TLR-4-MAPK/NF-κB pathway triggered by TLR-4 would be inhibited by TAK-242. Pyrrolidinecarbodithioic acid (PDTC) is a specific NF-κB inhibitor which can permeate the cell membranes and inhibit the activation of NF-κB. In order to confirm further that rTsgal binding to TLR-4 in Caco-2 cells activates MAPK-NF-κB pathway, two inhibitors TAK-242 and PDTC were used in the present study [45].

The storage solution of TAK-242 and PDTC was first prepared according to the manuals: 200 µg TAK-242 powder was dissolved in 11.055 µL dimethyl sulfoxide (DMSO) to be a storage solution with the concentration of 50 mM, and 16.429 mg PDTC powder is directly dissolved in 1 mL PBS to make a storage solution with a concentration of 100 mM. CCK-8 test was conducted to ascertain the cell viability after starvation treatment with 5, 10, 20 and 25 µM TAK-242 for 2 h before incubation with rTsgal, IIL CAg and LPS for 0.5 h [46]. Caco-2 cells were also treated with PDTC (100, 200, 300, 400, 500 mM) for 2 h, then incubated with rTsgal, IIL CAg and LPS for 0.5 h, and Western blot was performed to analyze the expression level of p-ERK1/2, ERK1/2, p-NF-κB p65 and NF-κB p65.

To investigate TAK-242 and PDTC inhibiting expression of TLR-4 in Caco-2 cells, the cells were respectively treated by 300 mM PDTC and 25 µM TAK-242 for 2 h, then, the cells were respectively incubated with rTsgal, IIL CAg and LPS for 24 h. The groups without TAK-242 were treated with DMEM containing 0.05% DMSO; while the groups without PDTC were treated with only DMEM. qPCR and Western blot were conducted to ascertain expression level of TLR-4 mRNA and protein in treated Caco-2 cells as previously reported.

### In vitro larval invasion assay

To assess the inhibitors’ suppressive role on larval invasion of enteral epithelium, the in vitro invasion assay was performed as previously described. Briefly, Caco-2 cell monolayer was first pretreated with 25 µM TAK-242 and 300 mM PDTC at 37 °C for 2 h. The muscle larvae were first activated into the IIL with 5% swine bile at 37 °C for 2 h, rTsgal and one hundred IIL were added to semisolid medium [10, 42]. Meanwhile, the PBS and PBS containing 0.05% DMSO were used as control. After being cultured at 5% CO_2_ at 37 °C for 2 h, the larvae invaded in Caco-2 cells was observed on light microscopy. The invaded IIL was a snake-like movement, and migrated within the cell monolayer, whereas non-invaded IIL were spirally coiled on the surface of cell monolayer.

### Challenge infection of mice with inhibitors-treated larvae

Sixty mice were randomly divided into 4 groups (15 animals each), each mouse was first treated with 100 µL PBS, PDTC (30 mg/kg), DMSO or TAK-242 (3 mg/kg) [47, 48]. Intraperitoneal injection was administrated once a day for consecutive 3 days. Then, all mice were orally challenged with 200 *T. spiralis* ML, and sacrificed at 6 days post-infection (dpi). Total RNAs from intestinal mucosa were isolated with TRIzol reagent (Invitrogen), and relative mRNA expression level of TLR-4, NF-κB p65, and cytokines (IL-1β, IL-6 and TGF-β) was ascertained by qPCR. Moreover, expression level of (IL-1β and TGF-β) in gut fluid from infected mice pretreated with TAK-242 and PDTC at 6 dpi was measured using an ELISA kit (Dakewe, Beijing, China). The intestines of infected mice were fixed in 4% formalin for 24 h and embedded in paraffin wax, 2-µm-thick tissue sections were prepared, deparaffinized and stained using hematoxylin and eosin (HE) stain [3, 35]. Pathological changes of intestinal mucosa in various groups of infected mice were observed under microscopy; the width of intestinal villus, number of Paneth cells per field (400×) were examined and numbered [28, 49]. Intestinal AWs were harvested from ten mice of each group at 6 dpi. The worm burden reduction was ascertained according to the number of intestinal enteral AWs recovered from inhibitor group compared to those from the solvent (DMSO or PBS) control group.

### Statistical analysis

SPSS 21.0 software was performed for analyzing the data in this study, and the data for larval invasion, protein expression levels, intestinal adult burden are presented as the mean ± standard deviation (SD). Shapiro–Wilk’s test and Levene’s test were used to check the datum’s normality and homogeneity, one-way ANOVA and Student’s *t* test were used to analyze inter-group differences. *P* < 0.05 was regarded as statistically significant.

## Results

### Expression and identification of rTsgal

**Figure 1:**
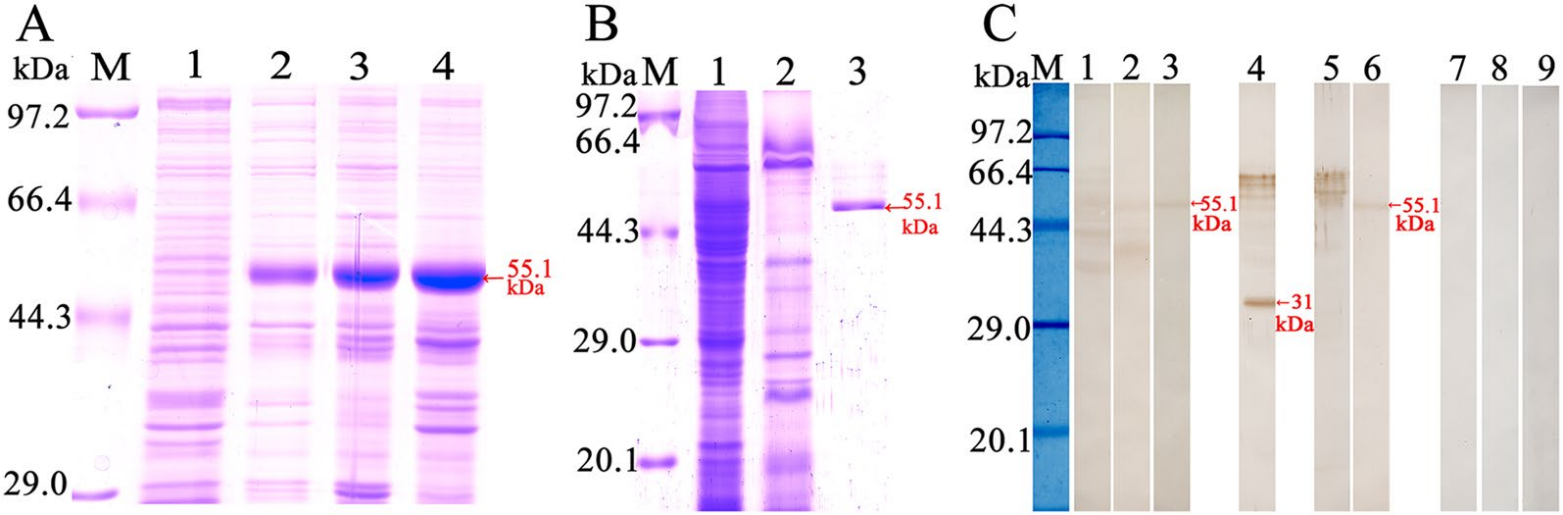
**Expression and identification of rTsgal. A** SDS-PAGE analysis of rTsgal, lane M: protein marker; Lane 1: Lysate of recombinant *E. coli* incorporating pGEX-4T-1/Tsgal prior to induction; Lane 2: Lysate of recombinant *E. coli* incorporating pGEX-4T-1/Tsgal following induction; Lane 3: rTsgal expressed in lysate supernatant of recombinant *E. coli* incorporating pGEX-4T-1/Tsgal following induction; Lane 4: rTsgal expressed in lysate precipitate of recombinant *E. coli* incorporating pGEX-4T-1/Tsgal following induction. **B** SDS-PAGE analysis of IIL antigens and purified rTsgal. Lane M: protein marker; lane 1: IIL crude antigens; lane 2: IIL ES antigens; lane 3: purified rTsgal. **C** Western blotting analysis of rTsgal antigenicity. Lane M: protein marker. IIL crude antigens (lane 1, 4) and rTsgal (lane 3, 6) were recognized by infection serum (lane 1, 3) and anti-rTsgal serum (lane 4, 6), but IIL ES antigens (lane 2, 5) were not recognized by infection serum (lane 2) and anti-rTsgal serum (lane 5). Lane 7–9: IIL crude, ES antigens and rTsgal were not recognized by normal serum.

SDS-PAGE results showed that rTsgal was expressed both in supernatant and precipitation, then the molecular weight (MW) of the rTsgal with GST tag purified by GST-Sefinose Resin 4FF was 55.1 kDa, which was consistent with the predicted MW of the rTsgal protein (rTsgal is 29.1 kDa, and the GST tag protein is 26 kDa) (Figure 1A and B).

To assess the humoral immune response triggered by rTsgal immunization, the titer of anti-rTsgal IgG at 2 weeks after last immunization was assayed by ELISA. The results showed that serum anti-rTsgal IgG titer reached 1:10^5^, demonstrating that rTsgal has good immunogenicity. Western blotting results revealed that rTsgal was recognized by anti-rTsgal serum and infection serum, but not by normal serum (Figure 1C). Moreover, anti-rTsgal serum and infection serum also recognized the natural Tsgal with 31 kDa in IIL crude antigens, but not in IIL ES antigens, suggesting that Tsgal was a worm somatic protein, but not a secretory protein.

### Caco-2 cell viability

The results of CCK-8 assay showed that when 10, 20, 30, 40 and 50 µg/mL rTsgal and 10, 20, 30, 40 µg/mL IIL crude antigens were co-cultured with Caco-2 cells for 24 and 48 h, the difference of cell viability among various dose groups of rTsgal and IIL crude antigens was no statistically different (rTsgal: *F*_24h_ = 0.384, *F*_48h_ = 1.583, *P* > 0.05; IIL crude antigens: *F*_24h_ = 1.296, *F*_48h_ = 1.226, *P* > 0.05), suggesting rTsgal and IIL crude antigens did not have obvious harm to the cell vitality (Additional file 1). Therefore, 50 µg/mL rTsgal and 20 µg/mL IIL crude antigens were used the subsequent experiment.

### Co-localization of rTsgal and TLR-4 in Caco-2 cell membrane

**Figure 2:**
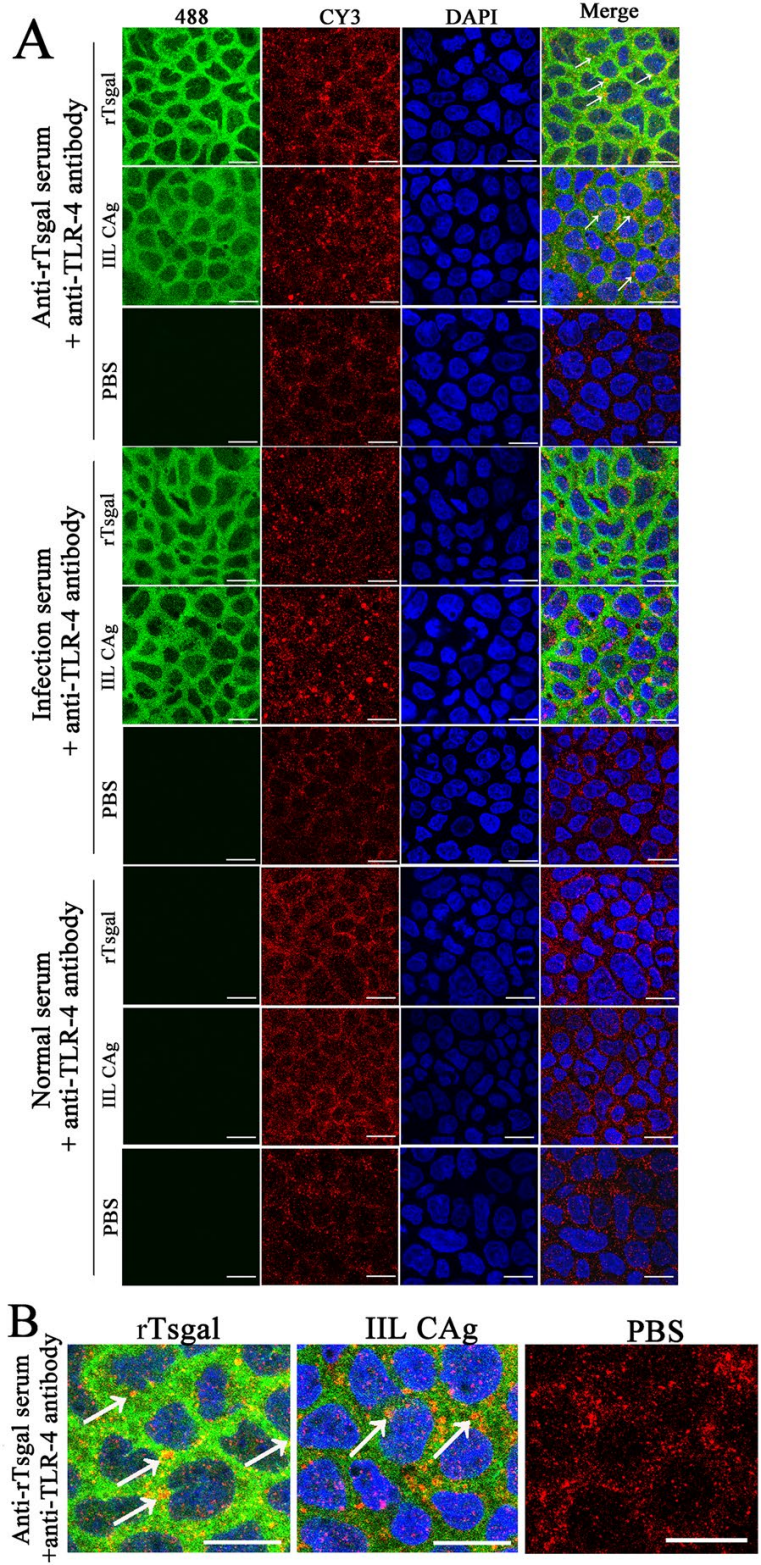
**Co-localization of rTsgal and TLR-4 on Caco-2 cell membrane. A** Caco-2 cells pre-incubated with rTsgal, IIL CAg and PBS were probed by mouse anti-rTsgal serum, infection serum and normal serum, and Alexa Fluor 488-conjugated anti-mouse IgG as secondary antibody. Caco-2 cells pre-incubated with rTsgal, IIL CAg and PBS were also probed by rabbit anti-TLR-4 antibody, and Cy3-conjugated anti-rabbit IgG was served as the secondary antibody. DAPI: cell nucleus was stained by DAPI as blue. **B** Enlarged areas of co-localization of rTsgal and TLR-4 on Caco-2 cells. The binding of rTsgal and TLR-4 was co-localized mainly on the cellular membrane as shown as white arrows. Scale bars: 40 μm.

The IIF results showed that specific green immunofluorescence on the surface of Caco-2 cells incubated with rTsgal and IIL CAg was detected by using anti-rTsgal serum and infection serum but not by normal mouse serum. And simultaneously, specific red immunofluorescence on the surface of Caco-2 cells incubated with rTsgal and IIL CAg was detected by anti-TLR-4 antibody. After being merged, the co-localization of rTsgal and TLR-4 was observed as orange mainly on the cellular membrane (Figure 2).

### Binding between rTsgal and TLR-4 detected by GST pull-down

**Figure 3:**
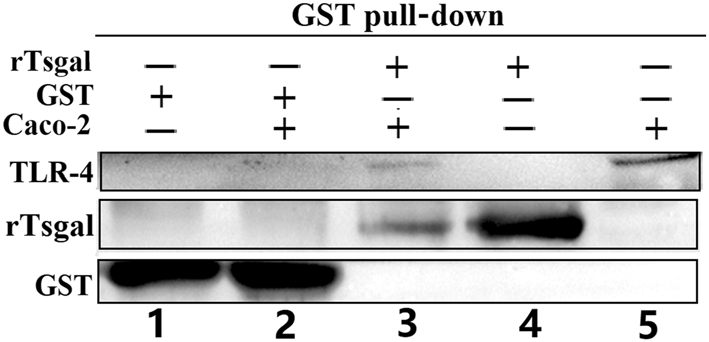
**Interaction between rTsgal and TLR-4 assayed by GST pull-down.** 1: GSH affinity resin combined with GST; 2: GSH affinity resin combined with GST and Caco-2 cell protein; 3: GST affinity resin combined with rTsgal with GST tag and Caco-2 protein; 4: GSH affinity resin combined with rTsgal with GST tag; 5: Caco-2 protein containing TLR-4.

The results of GST pull-down test showed that binding of GST tag with Caco-2 cell proteins was not detected, indicating that there was no specific interaction between GST and TLR-4 in Caco-2 cells. After rTsgal with GST tag was incubated with GST affinity resin, the binding of rTsgal and TLR-4 was observed (Figure 3, lane 3), indicating that there was a specific interaction between rTsgal and TLR-4 in Caco-2 cells.

#### Binding of rTsgal and TLR-4 assayed by Co-IP

**Figure 4:**
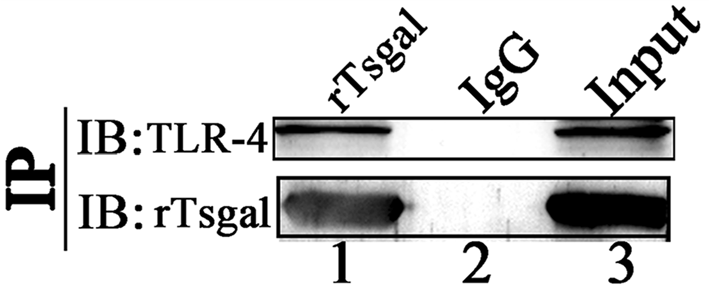
**Binding between rTsgal and TLR-4 detected by Co-IP.** rTsgal were first incubated with Caco-2 cells. Then, anti-rTsgal antibodies and protein A/G were added and incubated for 12 h. The bound proteins were subjected to SDS-PAGE and transferred to a NC membrane. The membrane was probed with anti-rTsgal antibodies and anti-TLR-4 antibody. Lane 1: pulled down immune complex (rTsgal, anti-rTsgal antibodies and TLR-4 in Caco-2 cell proteins); Lane 2: normal mouse IgG; Lane 3: Caco-2 cell proteins.

The binding of rTsgal and TLR-4 in Caco-2 cells was investigated by Co-IP and Western blotting. The results clearly revealed that rTsgal bound to TLR-4 and that the binding complex was pulled down by anti-rTsgal antibodies (Figure 4). No TLR-4 was pulled down by normal mouse IgG, indicating that rTsgal bound specifically to receptor TLR-4 in Caco-2 cells.

### TLR-4 expression level in Caco-2 cells incubated with rTsgal

**Figure 5:**
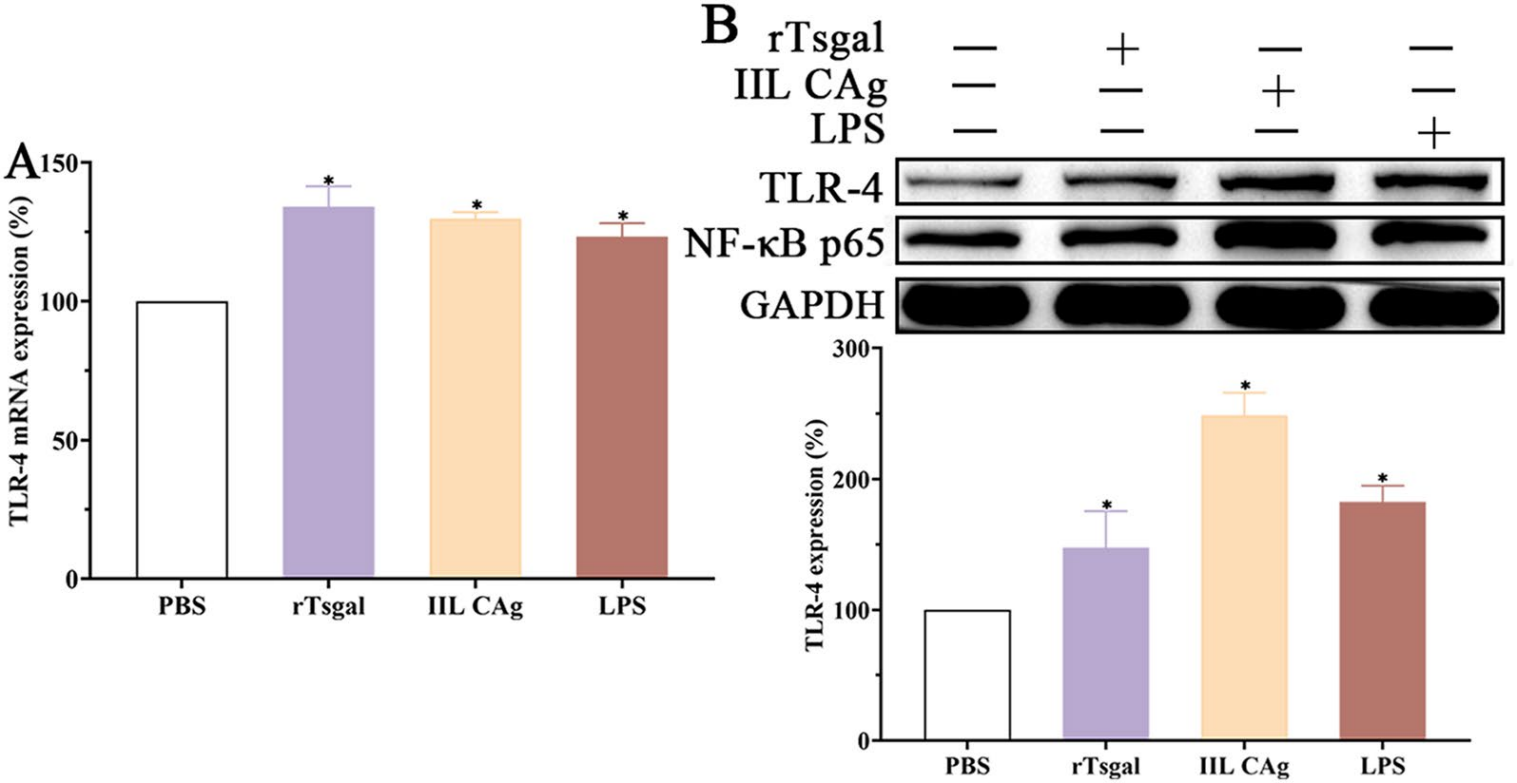
**rTsgal up-regulated TLR-4 expression level in Caco-2 cells. A** TLR-4 transcription level of Caco-2 cells incubated with rTsgal, IIL CAg and LPS. **B** TLR-4 expression level of Caco-2 cells incubated with rTsgal, IIL CAg and LPS. TLR-4 expression level of Caco-2 cells was obviously up-regulated after rTsgal binds to TLR-4 receptor in the cells. *P < 0.01 compared to the PBS group.

qPCR results showed that after Caco-2 cells were incubated with rTsgal (50 µg/mL), IIL CAg (20 µg/mL) and LPS (0.5 µg/mL) for 48 h, TLR-4 transcription level in Caco-2 cells was increased by 18.44, 34.15 and 29.85%, respectively, compared to the PBS group (*F* = 6.969, *P* < 0.05); TLR-4 expression level of treated Caco-2 cells was increased by 47.66, 148.60 and 82.57%, respectively (*F* = 38.19, *P* < 0.0001), suggesting that binding of rTsgal and TLR-4 in Caco-2 cells activated and up-regulated the TLR-4 expression level (Figure 5).

### Binding of rTsgal and TLR-4 activated MAPK-NF-κB signaling pathway

**Figure 6:**
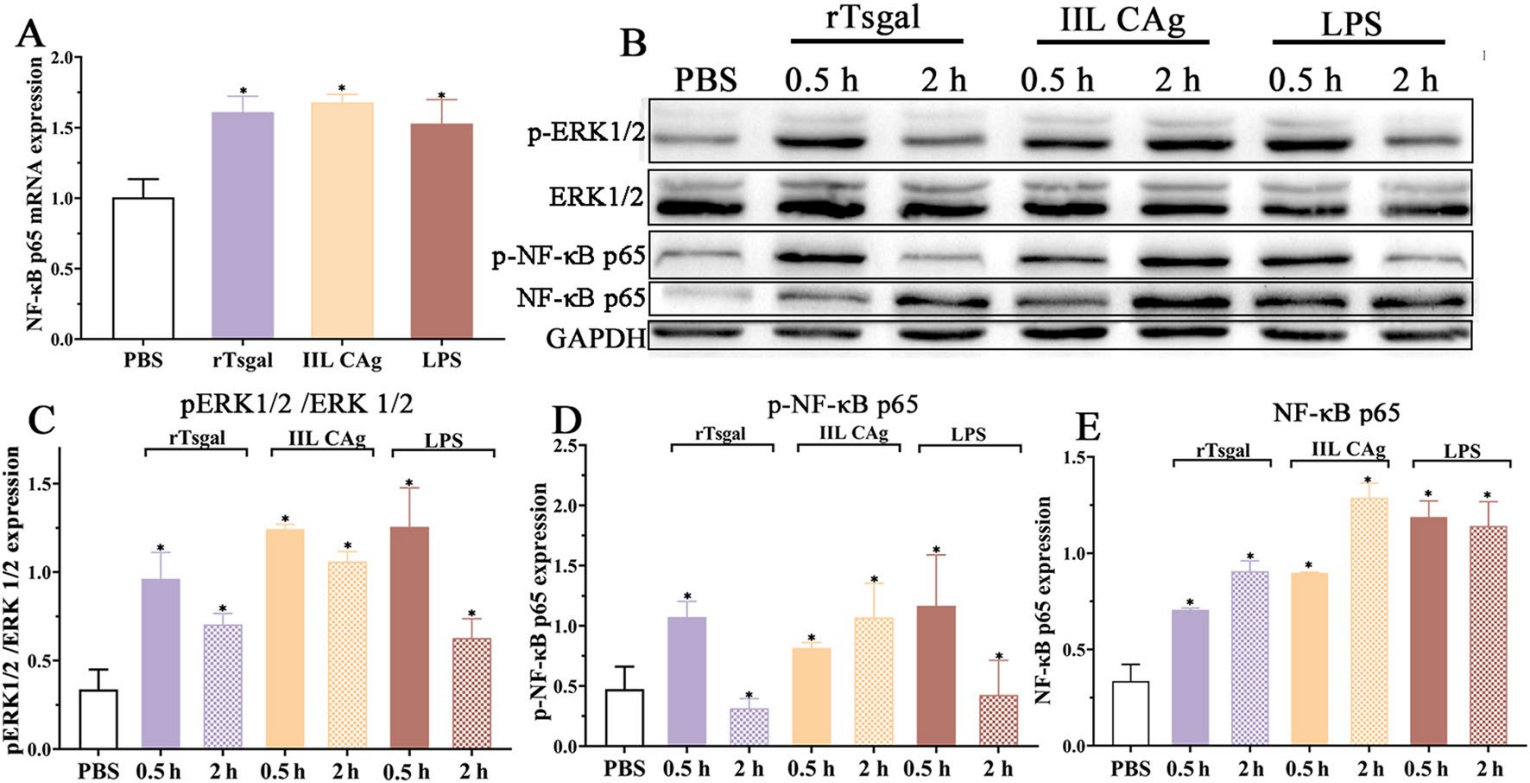
**Expression level of p-ERK1/2 and p-NF-κB p65 in Caco-2 cells incubated with rTsgal. A** NF-κB p65 mRNA expression level in Caco-2 cells incubated with rTsgal at 48 h. **B** Western blotting analysis of p-ERK1/2 and p-NF-κB p65 in Caco-2 cells incubated with rTsgal at 0.5 and 2 h. **C** Expression of p-ERK1/2 in Caco-2 cells incubated with rTsgal at 0.5 and 2 h. **D** Expression of p-NF-κB p65 in Caco-2 cells incubated with rTsgal at 0.5 and 2 h. **E** Expression of NF-κB p65 in Caco-2 cells incubated with rTsgal at 0.5 and 2 h. *P < 0.01 compared to the PBS group.

Compared to the PBS group, the NF-κB p65 transcription level of Caco-2 cells treated with rTsgal, IIL CAg and LPS at 48 h was increased by 60.22, 66.86 and 52.11%, respectively (*F* = 18.265, *P* < 0.01) (Figure 6A). Western blotting results showed that when Caco-2 cells were stimulated by rTsgal, and LPS for 0.5 h, the expression of p-ERK1/2 and p-NF-κB p65 were up-regulated compared to the PBS group (*F* = 23.829, *P* < 0.0001; *F* = 6.585, *P* < 0.01) (Figure 6B–D), but the expression level of total ERK1/2 expression level was not significantly changed. After stimulation for 2 h, p-ERK1/2 and p-NF-κB p65 expression began to decrease, but was similar to or higher than the PBS group. In group of Caco-2 cells incubated with IIL CAg at 0.5 and 2 h, p-ERK1/2 and p-NF-κB p65 expression was higher than the PBS group (*F*_0.5 h_ = 23.829, *F*_2h_ = 6.585, *P* < 0.01). NF-κB p65 expression of Caco-2 cells incubated with rTsgal, IIL CAg and LPS at 0.5 and 2 h was also increased compared to the PBS group (*F* = 10.948, *P* = < 0.01) (Figure 6E). The results showed that when rTsgal bound to TLR-4 on Caco-2 cells, MAPK-NF-κB signaling pathway was activated, NF-κB p65, p-NF-κB p65 and p-ERK1/2 were up-regulated, suggesting that rTsgal had the ability of binding to TLR-4, subsequently activating MAPK-NF-κB pathway.

### Cytokine transcription level in Caco-2 cells treated with rTsgal

**Figure 7:**
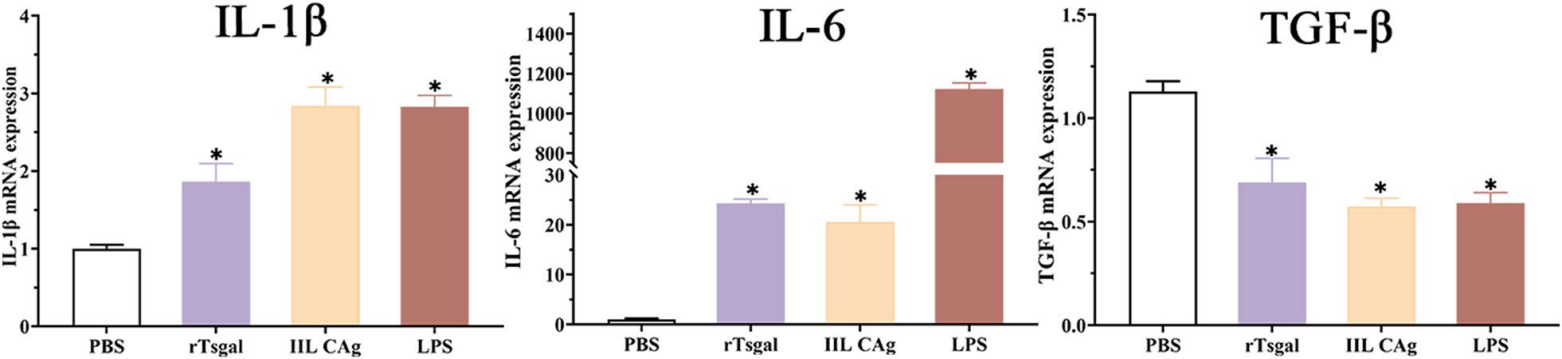
**Transcription level of IL-1β, IL-6 and TGF-β in Caco-2 cells treated with rTsgal.** After Caco-2 cells were incubated with rTsgal, the transcription level of IL-1β and IL-6 significantly increased, whereas TGF-β transcription level prominently reduced compared to the PBS group. *P < 0.0001 compared to the PBS group.

Compared to the PBS group, transcription level of IL-1β and IL-6 in Caco-2 cells treated with rTsgal evidently increased (*F*_IL-1β_ = 61.787, *F*_IL-6_ = 109.48, *P* < 0.0001), while TGF-β transcription level decreased obviously (*F* = 39.885, *P* < 0.0001) (Figure 7), indicating that rTsgal activated MAPK-NF-κB pathway via binding with TLR-4, and promoted the secretion of pro-inflammatory cytokine (IL-1β and IL-6) of Caco-2 cells.

### Inhibitors inhibited expression of rTsgal-activated MAPK/NF-κB pathway in Caco-2 cells

To ascertain whether TLR-4 and p-NF-κB p65 inhibitors inhibit the expression of MAPK/NF-κB pathway in Caco-2 cells, the optimal dose of TLR-4 and p-NF-κB p65 inhibitors (TAK-242 and PDTC) was first identified. Caco-2 cells were treated with various doses of TAK-242 (5, 10, 20, 25 µM) and PDTC (100, 200, 300, 400, 500 mM) for 2 h, and cell viability was measured by CCK-8 test, the result revealed that diverse dose of TAK-242 (0–25 µM) did not have evidently effects on Caco-2 cell viability (*F =* 1.273, *P* > 0.05), and 100–300 mM PDTC did not affect cell viability (*F* = 3.629, *P* > 0.05); but 400 and 500 mM PDTC decreased the cell viability (*F* = 68.884, *P* < 0.0001) (Additional file 2). Thereby, 25 µM TAK-242 and 300 mM PDTC were used in the following tests.

**Figure 8:**
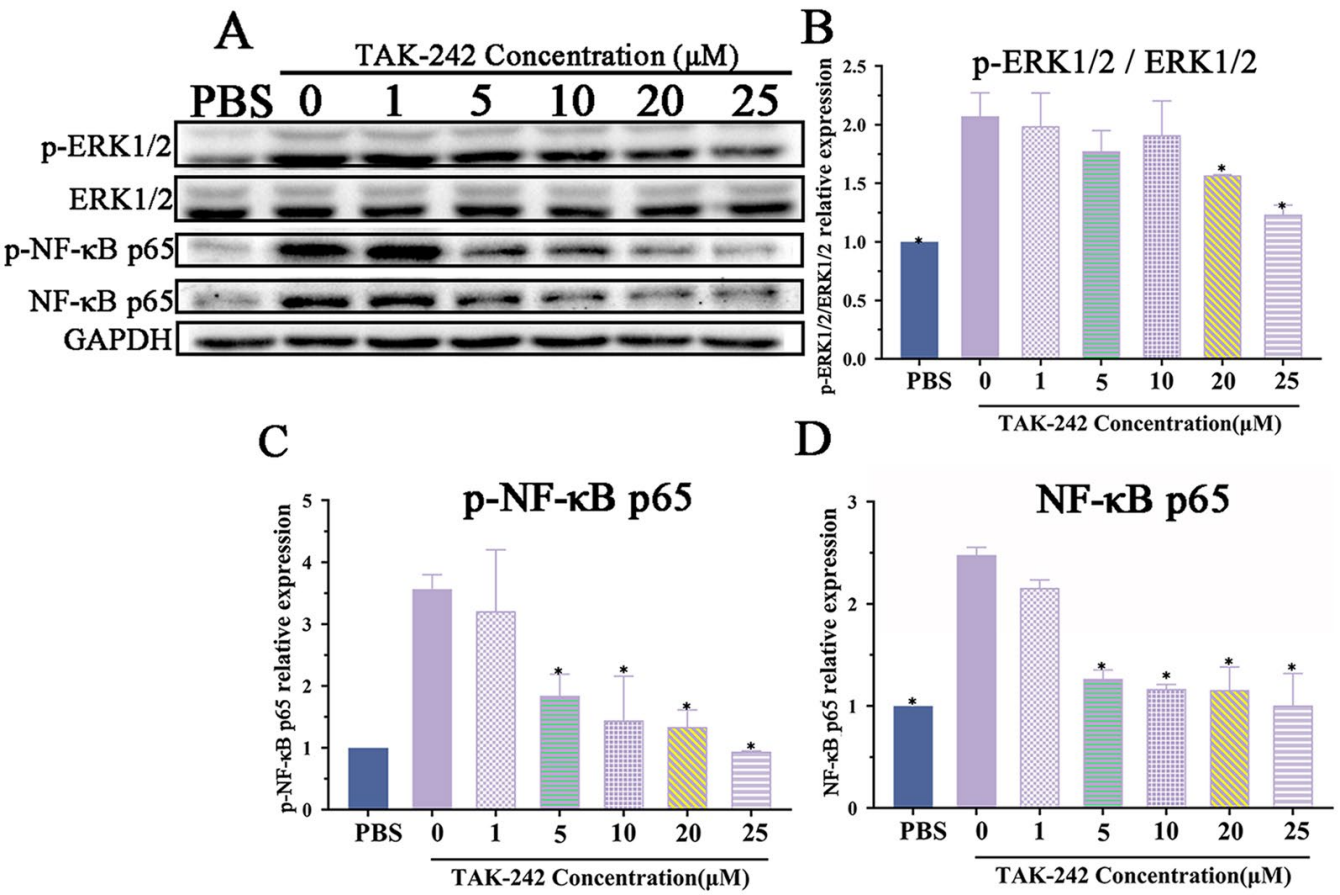
**Various dose of TAK-242 inhibited the rTsgal-activated MAPK/NF-κB pathway.** Caco-2 cells were first treated with TLR-4 inhibitor TAK-242 (1–25 µM) for 2 h, and then incubated with rTsgal, IIL CAg and LPS for 0.5 h, and Western blot was performed to analyze the expression level of p-ERK1/2, p-NF-κB p65 and NF-κB p65. **A** Western blotting of NF-κB pathway proteins of Caco-2 cells treated with TAK-242. **B** Expression of p-ERK1/2 in TAK-242 treated-Caco-2 cells. Expression of rTsgal-activated p-NF-κB p65 (**C**) and NF-κB p65 (**D**) in Caco-2 cells was inhibited by various dose of TAK-242. *P < 0.0001 compared to the PBS group.

Western blot results revealed that 1–10 µM TAK-242 did not inhibit the expression of p-ERK1/2, but 20 and 25 µM TAK-242 statistically inhibited the up-regulation of p-ERK1/2 compared to the solvent DMSO group (*F* = 14.085, *P* < 0.0001) (Figure 8A and B). The expression of p-NF-κB p65 and NF-κB p65 was obviously suppressed by 5–25µM TAK-242 (p-NF-κB p65: *F* = 20.861, *P* < 0.0001; NF-κB p65: *F* = 44.314, *P* < 0.0001), compared to the PBS group (Figure 8C and D). Therefore, to assess effect of TAK-242 on MAPK/NF-κB pathway in Caco-2 cells, 25 µM TAK-242 was used in the subsequent experiments.

**Figure 9:**
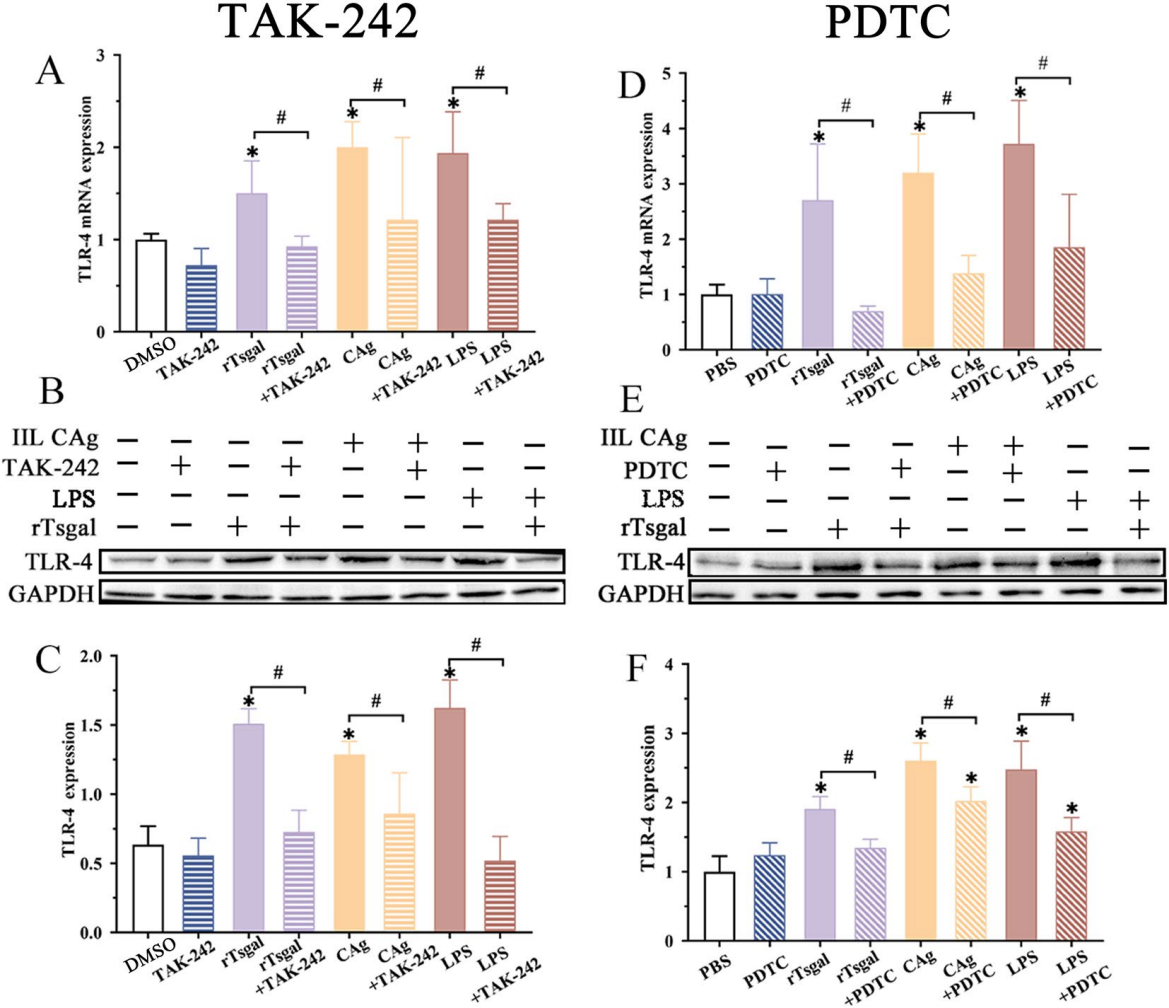
**TAK-242 and PDTC abrogated rTsgal-activated TLR-4 expression in Caco-2 cells. A**, **D** qPCR analysis of rTsgal–activated TLR-4 mRNA expression in Caco-2 cells treated TAK-242 (**A**) and PDTC (**D**). **B**, **E** Western blot analysis of expression of rTsgal–activated TLR-4 protein expression in Caco-2 cells treated with TAK-242 (**B**) and PDTC (**E**). **C**, **F** comparison of rTsgal–activated TLR-4 protein expression in various groups of Caco-2 cells treated with TAK-242 (**C**) and PDTC (**F**). TAK-242 and PDTC distinctly abrogated the rTsgal-activated TLR-4 expression in Caco-2 cells. *P < 0.05 compared to the solvent (DMSO or PBS) group. ^#^*P* < 0.05 compared to between the two groups.

qPCR results showed that rTsgal, IIL CAg and LPS obviously increased the expression of TLR-4 mRNA in Caco-2 cells, compared to the PBS group; however, the expression of TLR-4 mRNA significantly decreased in TAK-242 treated cells, compared to the groups without TAK-242 (Figure 9A) (*F* = 3.179, *P* < 0.05). Western blot results revealed that the expression of TLR-4 protein in Caco-2 cells treated with rTsgal, IIL CAg and LPS was evidently increased, but TLR-4 protein expression obviously reduced following TAK-242 use (Figure 9B, C) (*F* = 7.627, *P* < 0.05). Moreover, NF-κB specific inhibitor PDTC also evidently inhibited rTsgal-activated TLR-4 mRNA expression in Caco-2 cells (Figure 9D) (*F* = 9.201, *P <* 0.0001); rTsgal-activated TLR-4 protein expression in Caco-2 cells was obviously suppressed and abrogated by PDTC (Figure 9E, F) (*F* = 18.276, *P* < 0.0001). The results demonstrated that both of TLR-4 receptor specific inhibitor TAK-242 and NF-κB specific inhibitor PDTC distinctly abrogated the rTsgal-activated the TLR-4 expression in Caco-2 cells.

### TAK-242 and PDTC inhibited rTsgal activated-NF-κB pathway and cytokine response

**Figure 10:**
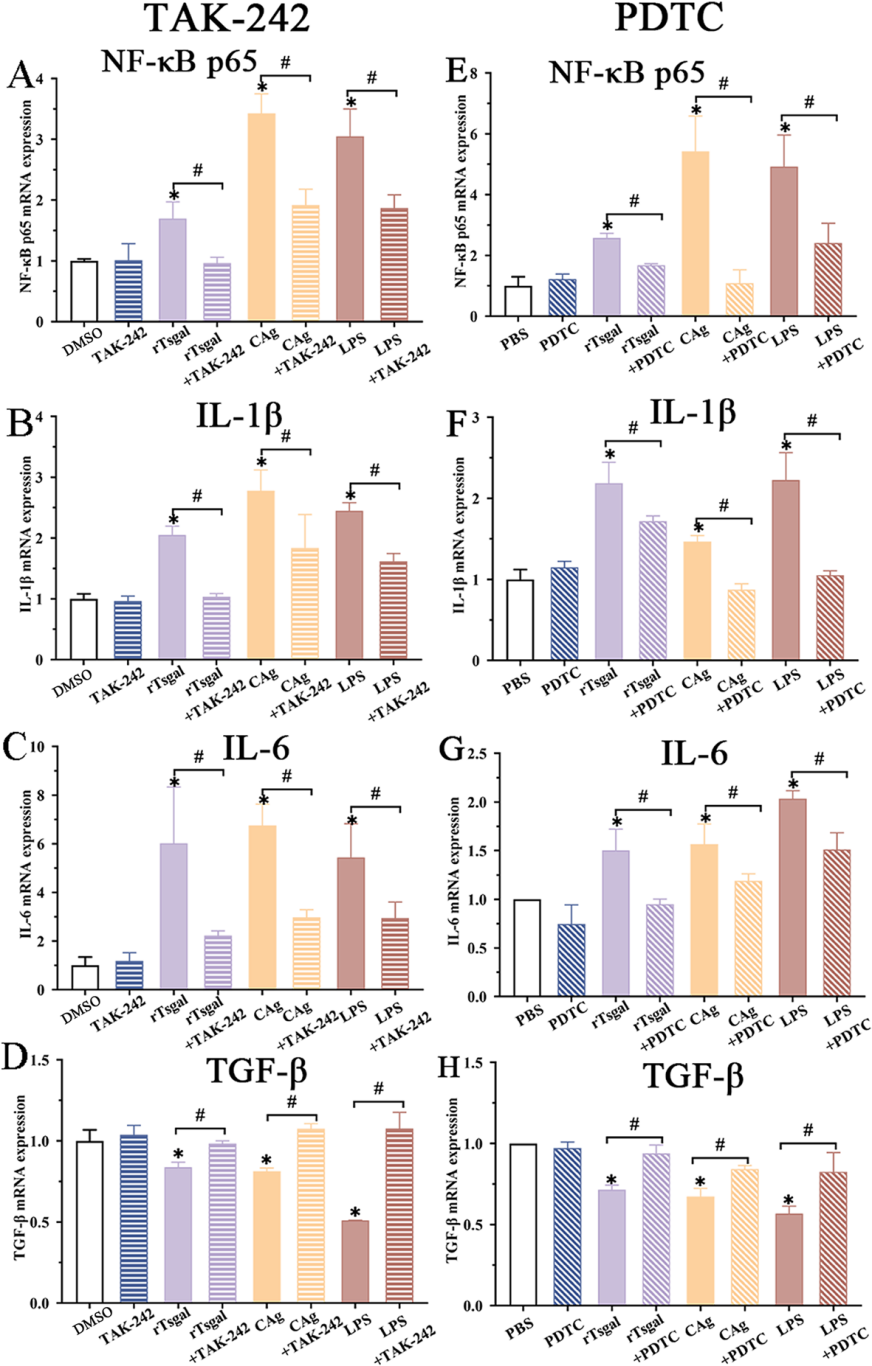
**TAK-242 and PDTC inhibited rTsgal-activated transcription of NF-κB pathway and cytokines in Caco-2 cells. A**, **E** qPCR analysis of rTsgal-activated expression of NF-κB p65 mRNA in Caco-2 cells treated with TAK-242 (**A**) and PDTC (**E**). **B**–**D** and **F**–**H** qPCR analysis of rTsgal**-**activated expression of IL-1β (**B**, **F**), IL-6 (**C**, **G**) and TGF-β (**D**, **H**) mRNA in Caco-2 cells treated with TAK-242 (**B**–**D**) and PDTC (**F**–**H**). *P < 0.0001 compared to the DMSO or PBS control group. ^#^*P* < 0.05 compared to between the two groups.

Caco-2 cells were treated with 25 µM TAK-242 and 300 mM PDTC for 2 h, then incubated with rTsgal, IIL CAg and LPS for 48 h. qPCR was conducted to assess the transcription level of NF-κB p65 and cytokines (IL-1β, IL-6 and TGF-β). qPCR results showed that the rTsgal up-regulated transcription level of NF-κB p65 in Caco-2 cells was evidently abrogated by TAK-242 and PDTC (Figure 10A and E) (*F*_TAK-242_ = 36.07, *F*_PDTC_ = 42.59, *P <* 0.0001). rTsgal up-regulated transcription of pro-inflammatory cytokines (IL-1β and IL-6) was also decreased and depressed by TAK-242 (*F*_IL-1β_ = 23.273, *F*_IL-6_ = 13.31, *P <* 0.0001) (Figure 10B and C) and PDTC (*F*_IL-1β_ = 31.75, *F*_IL-6_ = 23.902, *P <* 0.0001) (Figure 10F and G). However, rTsgal down-regulated transcription of anti-inflammatory cytokine (TGF-β) was increased by TAK-242 (*F*_TGF-β_= 42.755, *P <* 0.0001) and PDTC (*F*_TGF-β_ = 23.747, *P <* 0.0001) (Figure 10D and H). The results suggested that the two inhibitors TAK-242 and PDTC significantly suppressed rTsgal activated NF-κB pathway and expression of pro-inflammatory cytokines (IL-1β, IL-6), but up-regulated expression of anti-inflammatory cytokine (TGF-β).

**Figure 11:**
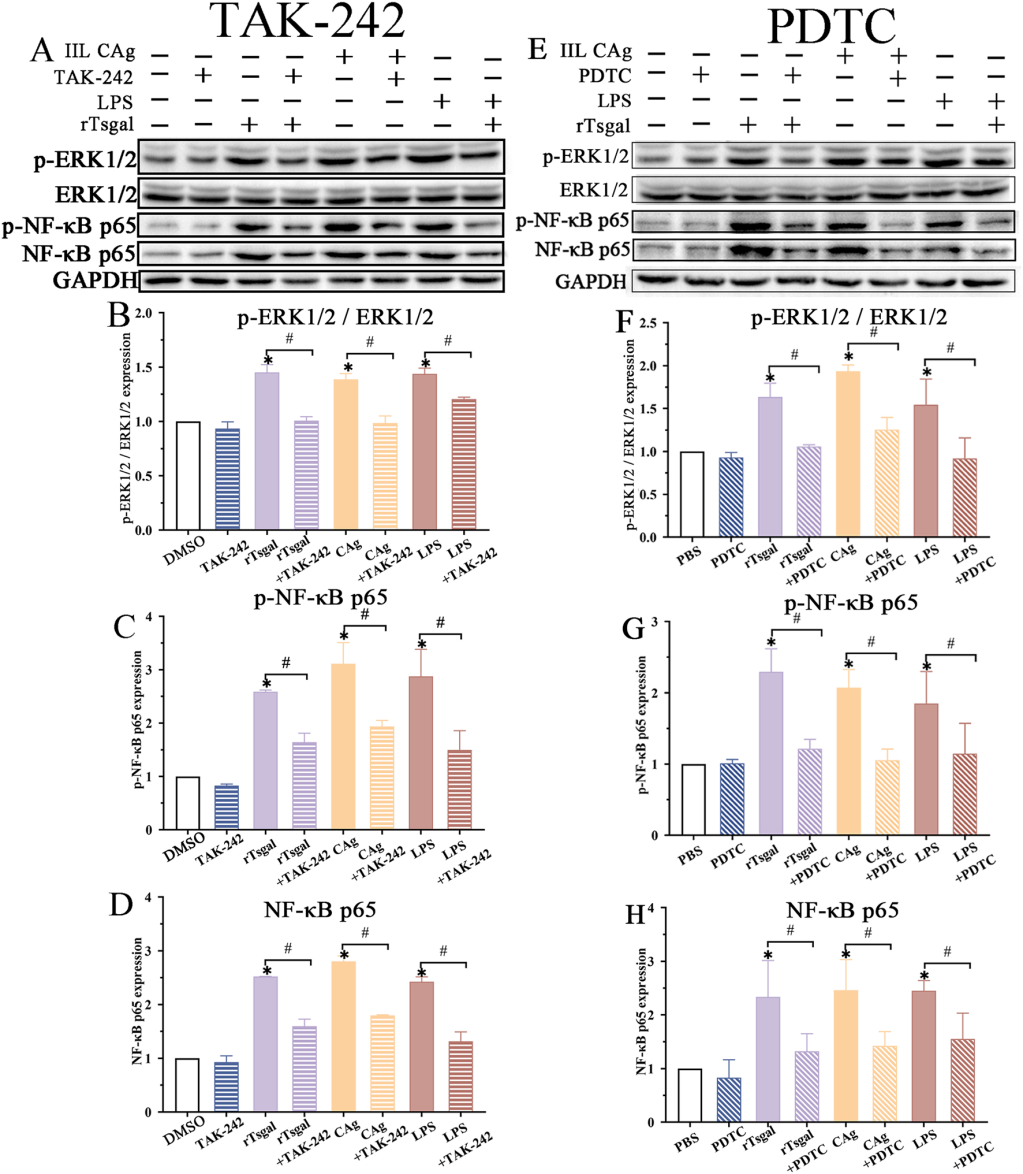
**TAK-242 and PDTC inhibited rTsgal-activated expression of NF-κB pathway proteins in Caco-2 cells. A**, **E** Western blot analysis of rTsgal-activated expression of NF-κB p65 protein in Caco-2 cells treated with TAK-242 (**A**) and PDTC (**E**). **B**–**D** and **F**-**H** Western blot analysis of rTsgal-activated expression of p-ERK1/2 (**B**, **F**), p-NF-κB p65 (**C**, **G**) and NF-κB p65 (**D**, **H**) in Caco-2 cells treated with TAK-242 (**B**–**D**) and PDTC (**F**–**H**). *P < 0.0001 compared to the DMSO or PBS control. ^#^*P* < 0.05 compared between the two groups.

Furthermore, western blotting results showed that compared to the alone rTsgal group, the up-regulated expression of rTsgal-activated p-ERK1/2 was obviously inhibited by TAK-242 (*F* = 57.587, *P <* 0.0001) (Figure 11A and B) and PDTC (*F* = 17.130, *P <* 0.0001) (Figure 11E and F). The up-regulation of rTsgal-induced p-NF-κB p65 and NF-κB p65 was also significantly reduced by TAK-242 (*F*_p-p65_ = 29.689, *F*_p65_ = 216.79, *P <* 0.0001) (Figure 11C, D), and PDTC (*F*_p-p65_ = 11.243, *F*_p65_ = 7.723, *P <* 0.0001) (Figure 11G and H). The results indicated that TLR-4 inhibitor TAK-242 and NF-κB inhibitor PDTC significantly inhibited the rTsgal-activated MAPK/NF-κB pathway and reduced the expression levels of p-ERK1/2 and p-NF-κB p65 in Caco-2 cells.

### TAK-242 and PDTC inhibited the larval invasion of Caco-2 cells

**Figure 12:**
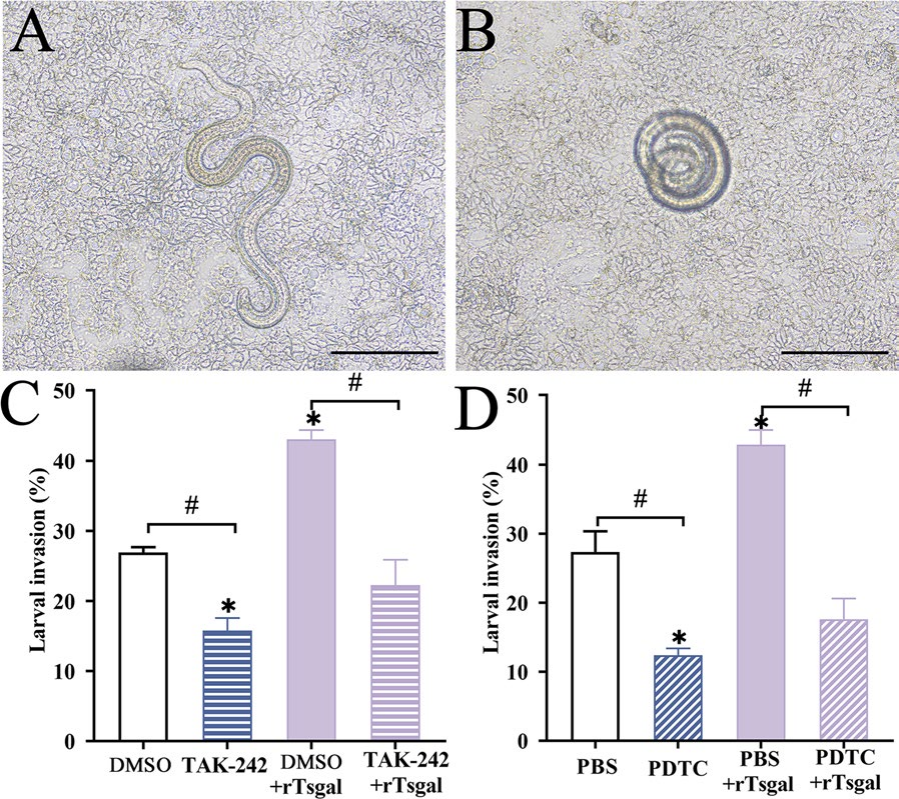
**TAK-242 and PDTC inhibited the larval invasion of Caco-2 cells.** Caco-2 monolayer was first pretreated with 25 µM TAK-242 and 300 mM PDTC at 37 °C for 2 h. The muscle larvae were activated into the IIL using 5% swine bile for 2 h at 37 °C, then added onto the Caco-2 monolayer and larval invasion was examined under microscope at 2 h after co-culture. **A** The invaded larva was locomotive and migrating in the monolayer. **B** Non-invaded larva was coiled on the cell surface. **C**, **D** TAK-242 (**C**) and PDTC (**D**) inhibited the larval invasion. The results are expressed as the mean ± SD of three independent tests. Scale bars: 20 μm. *P < 0.05 compared to the DMSO or PBS group. ^#^*P* < 0.05 compared between the two groups.

As shown in Figure 12A, the invaded larva was locomotive and wriggly, and non-invaded larvae were spirally coiled on the surface of Caco-2 monolayer (Figure 12B). When rTsgal was added into medium, rTsgal obviously facilitated larval invasion of Caco-2 cells, compared to the only PBS group (*χ*^2^ = 4.947, *P <* 0.05) and the PBS + DMSO control group (*χ*^2^ = 4.660, *P <* 0.05). However, larval invasion was respectively inhibited by TAK-242 (*χ*^2^ = 51.224, *P <* 0.0001) and PDTC (*χ*^2^ = 6.08, *P <* 0.05) (Figure 12C and D). Moreover, the facilitative role of rTsgal on larval invasion was also abrogated by TAK-242 (χ^2^ = 8.728, *P <* 0.05) and PDTC (*χ*^2^ = 13.008, *P <* 0.0001). The results indicated that inhibitors of TLR-4 receptors and NF-κB pathway significantly impeded the larval invasion of gut epithelium, and further suggested that rTsgal binding to TLR-4 in gut epithelium activated NF-κB p65 pathway, mediated larval invasion of gut mucosa, promoted the secretion of pro-inflammatory cytokine (IL-1β and IL-6) and resulted intestinal inflammation.

### TAK-242 and PDTC inhibited larval invasion and reduced gut adult burden by blocking TLR-4 and NF-κB p65 pathway

**Figure 13:**
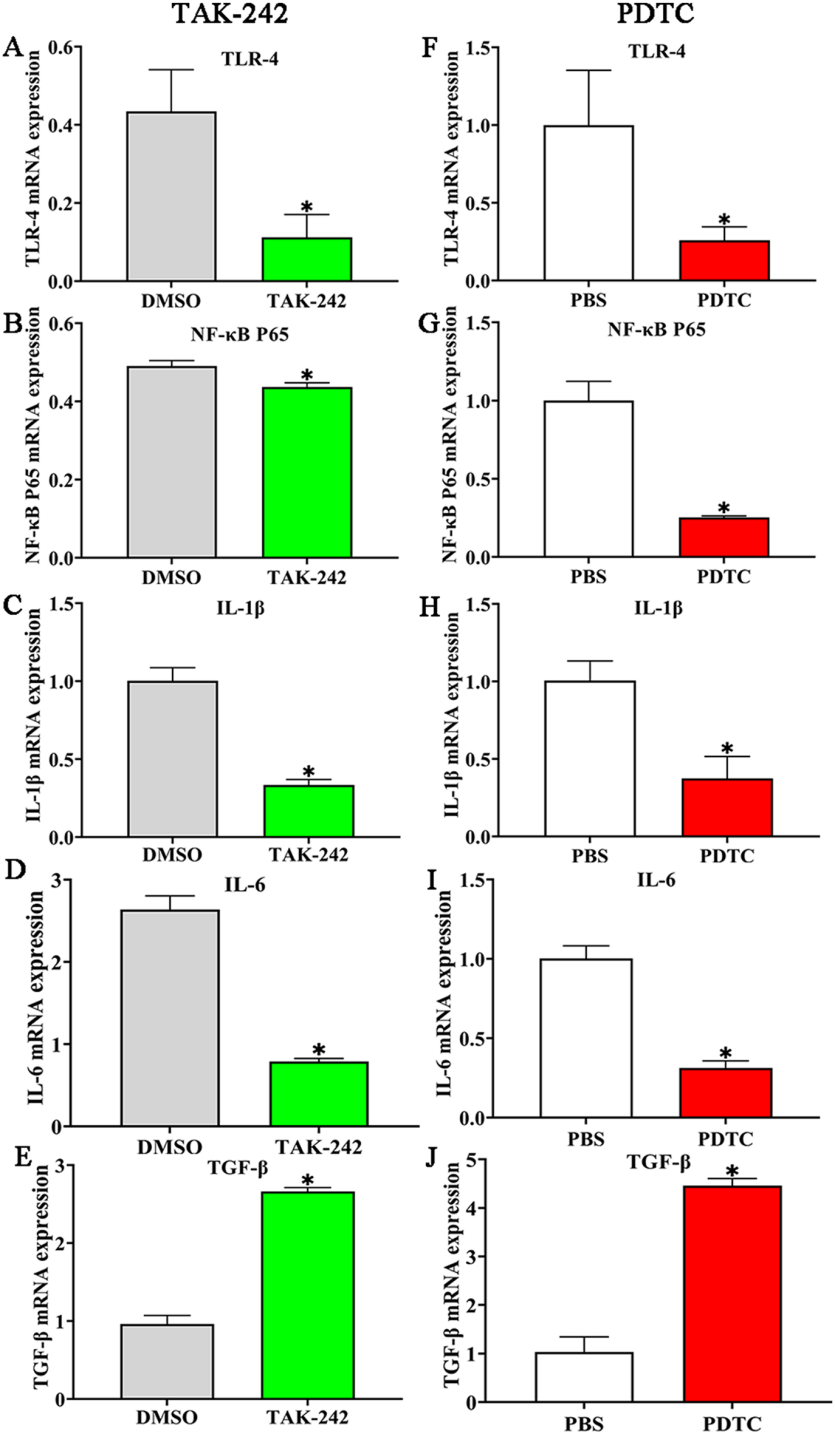
**TAK-242 and PDTC inhibited transcription of TLR-4, NF-κB p65, and IL-1β and IL-6 in infected murine intestines. A**, **F** qPCR analysis of TLR-4 mRNA transcription level in TAK-242 (**A**) and PDTC (**F**) group of infected mice. **B**, **G** qPCR analysis of NF-κB mRNA transcription in the TAK-242 (**B**) and PDTC (**G**) group of infected mice; **C**–**E** qPCR analysis of mRNA expression levels of IL-1β (**C**), IL-6 (**D**) and TGF-β (**E**) in infected mice pretreated with TAK-242; **H**–**J** qPCR analysis of mRNA expression levels of IL-1β (**H**), IL-6 (**I**) and TGF-β (**J**) in PDTC-pretreated infected mice. Statistical differences are marked with asterisks (*) compared to its solvent group at *P* < 0.05.

qPCR results revealed that TLR-4 specific inhibitor TAK-242 significantly reduced the TLR-4 transcription level in gut mucosa of infected mice at 6 dpi, compared to the DMSO group (*t*_TLR-4_ = 4.576, *P* < 0.05) (Figure 13A), the NF-κB p65 transcription level was also inhibited by TAK-242 (*t*_NF-κB_=5.252, *P* < 0.05) (Figure 13B). TAK-242 also distinctly suppressed the transcription levels of pro-inflammatory cytokines (IL-1β and IL-6) in infected mice (*t*_IL-1β_ = 12.739, *t*_IL-6_ = 18.714, *P* < 0.0001) (Figure 13C and D), and evidently enhanced the transcription of anti-inflammatory cytokine (TGF-β), compared to the DMSO group (*t*_TGF-β _= − 17.237, *P <* 0.0001) (Figure 13E). Furthermore, the NF-κB p65 specific inhibitor PDTC notably reduced the transcription levels of TLR-4 and NF-κB p65, compared to the PBS group (*t*_TLR-4_ = 3.533, *P* < 0.05; *t*_NF-κB p65_ = 10.551, *P* < 0.001) (Figure 13F and G). Moreover, PDTC also distinctly decreased the transcription levels of pro-inflammatory cytokines (IL-1β and IL-6) in infected mice (*t*_IL-1β_ = 5.773, *P <* 0.01; *t*_IL-6_ = 13.050, *P* < 0.0001) (Figure 13H, I) and obviously up-regulated the transcription of anti-inflammatory cytokine (TGF-β) (*t* = − 24.278, *P* < 0.0001) (Figure 13J). The results showed that both TAK-242 and PDTC significantly inhibited the mRNA expression level of TLR-4 and NF-κB p65, reduced production of pro-inflammatory cytokines (IL-1β and IL-6) and increased secretion of anti-inflammatory cytokine (TGF-β) in infected mice, subsequently alleviated intestinal inflammation and impeded larval intrusion of gut mucosa.

**Figure 14:**
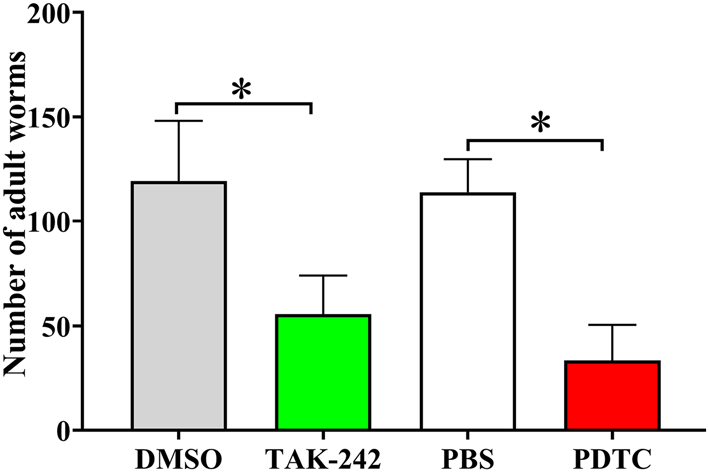
**TAK-242 and PDTC reduced intestinal adult burden of infected mice.** The data are presented as mean ± SD from ten mice of each group. *P < 0.0001 compared to its solvent (DMSO or PBS) control group.

Ten mice from each group of infected mice were euthanized at 6 dpi to investigate the suppressive effect of two inhibitors on *T. spiralis* invasion and infection; the number of AW were recovered from intestine and numbered, intestinal AW burdens were assessed. The results showed that compared to their solvent (DMSO or PBS) control group, the TAK-242 and PDTC groups exhibited a 40.21 and 31.86% reduction of intestinal AW burden, respectively (*χ*^2^_TAK-242_ = 486.618, *χ*^2^_PDTC_ = 288.729, *P <* 0.0001) (Figure 14). The results suggested that the specific inhibitors of TLR-4 receptor and MAPK/NF-κB pathway might impede the binding of Tsgal on the IIL surface to TLR-4 in gut epithelium and blocked activation of MAPK/NF-κB pathway and impeded larval invasion, and thereby decreased enteral adult burdens.

### TAK-242 and PDTC alleviated intestinal inflammation in infected mice

**Figure 15:**
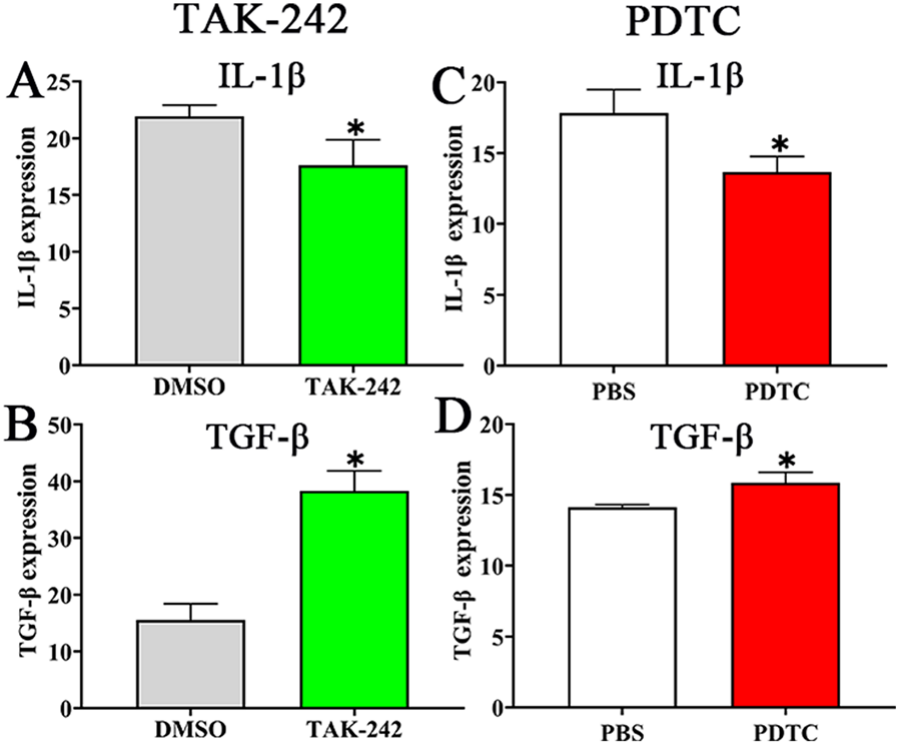
**Expression levels of IL-1β and TGF-β in infected murine intestines was detected by ELISA. A**, **C** IL-1β expression level was inhibited by TAK-242 (**A**) and PDTC (**C**). **B**, **D** TGF-β expression level was increased in TAK-242 (**B**) and PDTC (**D**) group of infected mice. The data are presented as mean ± SD from five mice of each group. *P < 0.05 compared to its solvent (DMSO or PBS) control group.

Intestinal pathological change of infected mice was observed at 6 dpi. In the solvent (DMSO and PBS) control groups, intestinal sections exhibited the destruction of villus structure, villous edema, and inflammatory cell infiltration in the villus, and increased number of Paneth cells and intracellular particles (Additional file 3). However, in the infected mice pretreated with TLR-4 specific inhibitor TAK-242 and NF-κB inhibitor PDTC, intestinal sections revealed that enteral villus width was obviously narrower than the solvent control groups (*t*_TAK-242_ = 3.250, *P <* 0.05; *t*_PDTC_ = 3.632, *P <* 0.05); the number of Paneth cells of two inhibitor groups was significantly less than the solvent control groups (*t*_TAK-242_ = 18.500, *P <* 0.0001; *t*_PDTC_ = 6.718, *P <* 0.01) (Additional file 4). Moreover, expression level of IL-1β and TGF-β in gut fluids of infected mice was measured by ELISA at 6 dpi. The results showed that compared to the solvent DMSO and PBS group, expression levels of pro-inflammatory cytokine (IL-1β) in infected mice pretreated with TAK-242 and PDTC was significantly decreased (*t*_TAK-242_ = 3.058, *t*_PDTC_ = 3.646, *P* < 0.05), while expression level of anti-inflammatory cytokine (TGF-β) in infected mice pretreated with TAK-242 and PDTC was evidently increased (*t*_TAK-242_ = − 8.741, *P* < 0.01; *t*_PDTC_ = − 3.954, *P* < 0.05) (Figure 15). The results demonstrated that TAK-242 and PDTC inhibited pro-inflammatory cytokine expression and restored production of anti-inflammatory cytokine in infected mice. Our results indicated that *T. spiralis* infection caused intestinal inflammatory reaction, whereas the TLR-4 receptor inhibitor and NF-κB inhibitor relieved and ameliorated intestinal inflammation, further suggested that Tsgal binding to TLR-4 activated MAPK-NF-κB pathway, induced intestinal inflammation and mediated larval invasion of gut mucosa.

## Discussion

Galectins as a class of glycoproteins that can bind glycans and agglutinate erythrocytes, have been widely studied in parasites and play a key role in the invasion and pathogenesis of parasites. The epidermis of *Angiostrongylus cantonensis* L5 larvae expressed galectin-1, resisted the harmful effects of host’s oxidative stress and facilitated the nematode parasitism [50], and galectin-2 secreted by *Brugia malayi* can modulate the host immune response and induce Th1 cell apoptosis [51]. Galectin of *Amoeba* protozoa can also promote its adhesion to the host colonic epithelium [23]. Galectin from *T. spiralis* (Tsgal) specifically bound to IECs and mediated larval intrusion into IECs, but the mechanism by which Tsgal mediates the invasion is not clear [14].

In our previous studies, rTsgal was ligated with expression vector pQE-80L and transformed into *E. coli* BL21 (DE3) for expression, but rTsgal carried His tag and had some limitations for functional study of Tsgal. To investigate further the interaction of Tsgal and host gut epithelium, the Tsgal was ligated with pMD-19-T and cloned into the pGEX-4T-1 expression vector with GST tag. The rTsgal with GST tag was recognized by anti-rTsgal serum and infection serum, demonstrating that rTsgal with GST tage has good immunoreactivity. Tsgal as a pathogen-associated pattern recognition molecule (PAMP) produced by *T. spiralis* larvae could bind to multiple pattern recognition receptors (PRRs) in intestine to trigger a cascade response in gut epithelium. TLR-4 is a more thoroughly studied cell membrane-associated PRR, which activated the NF-κB (nuclear transcription factor) signaling pathway in cells, leading to cell inflammation, autophagy and apoptosis [52]. In order to assess whether Tsgal results in such changes in Caco-2 cells, the interaction of rTsgal with TLR-4 in Caco-2 cells is investigated in this study.

Pull-down and Co-IP are the most useful techniques identifying the interactions between two proteins, such as the identification of the binding between two key factors in *Trypanosoma brucei* cell division, the flagella attachment region tip localization protein (FPRC), and the cytokinesis initiation factor CIF4; the interaction between the CRDs of *Amoeba* trophozoite and the colonic epithelium [23, 53]. In this study, the IIF results showed that TLR-4 was mainly distributed on the cellular membranes of Caco-2 cells. When rTsgal was incubated with Caco-2 cells, the co-localization of rTsgal and TLR-4 on the cytomembrane was observed. Therefore, the binding of rTsgal with TLR-4 was further investigated by pull-down and Co-IP. To exclude the impact of GST, a GST blank control was used. The results of pull-down and Co-IP confirmed the binding between rTsgal and TLR-4 in Caco-2 cells. The cell viability assay revealed that 50 µg/mL rTsgal and 20 µg/mL IIL crude antigens did not have obvious affects to the cell vitality. And 500 ng/mL LPS were used based on the reference on TLR-4 detection [54]. Therefore, the above-mentioned concentrations of rTsgal, IIL crude antigens and LPS were used the subsequent experiments.

In inflammatory bowel disease (IBD), TLR-4 in intestine induces a Th1 inflammatory response, producing the inflammatory cytokines IFN-γ and tumor necrosis factor (TNF), and then upregulating TLR-4, forming an “autocrine loop” [55]. When *T. spiralis* IIL invade gut epithelium and they trigger an evident gut mucosal inflammatory reaction, so it is assumed that the phenomenon of autocrine loop is also present at intestinal stage of *T. spiralis* infection [56]. Our results showed that TLR-4 expression was significantly upregulated after Caco-2 cells were incubated with rTsgal. Additionally, bacterial endotoxin could up-regulate cellular TLR-4 expression [57]. To exclude the effect of bacterial endotoxin in rTsgal on TLR-4 expression in Caco-2 cells, rTsgal was treated using polymyxin B to remove LPS in this study.

The well-studied inflammatory signaling pathway is the activation of the nuclear transcription factor NF-κB by TLR-4 into the cell nucleus, which leads to the production of downstream effectors such as inflammatory factors, cellular autophagy and apoptosis. NF-κB is a trimer composed of IκB, p50, and p65. p65 is activated into phosphorylated p65 and then enters the nucleus, causing the transcription and expression of various cytokines such as inflammatory factors and adhesion molecules, thus participating in the progression and development of several inflammatory diseases [58]. Previous studies demonstrated that TLR-4 and TLR-9 expression was up-regulated in intestine of *T. spiralis*-infected mice via the NF-κB pathway [18]. In the present study, to investigate whether binding of rTsgal to TLR-4 in Caco-2 cells triggers the NF-κB signaling pathway, transcription and expression level of NF-κB pathway proteins was assessed by qPCR and Western blot. The results revealed that NF-κB transcription levels were found to be significantly upregulated after Caco-2 cells incubating with rTsgal at 0.5 and 2 h. The expression level of pNF-κB p65 was up-regulated at 0.5 h and had begun to decrease to the levels of PBS control group at 2 h. It is likely because phosphorylation of NF-κB p65 is a transient process in response to stressful stimulation. Both transcription and expression levels of NF-κB p65 are up-regulated, in addition to phosphorylation, there might be methylation, ubiquitination, and acetylation, and these modifications collectively regulate the NF-κB pathway in both positive and negative directions [59]. Moreover, pERK1/2 was also significantly up-regulated after Caco-2 cells were treated with rTsgal for 0.5 h, and decreased at 2 h. But the ERK1/2 level was increased at 0.5 and 2 h compared to the PBS group. It has been found that MAPK promoted the synthesis of COX-2, iNOS and pro-inflammatory factors by controlling the activation of NF-κB [60], so the NF-κB activation by rTsgal binding to TLR-4 might be involved in the ERK1/2 pathway. rTsgal had the ability to bind with TLR-4 on cell membranes to stimulate the phosphorylation of ERK1/2. p-ERK1/2 could dissociate IκB, an inhibitor of NF-κB, from NF-κB tripolymer, releasing p50-p65 complex, leading to up-regulation of NF-κB p65 and pNF-κB p65, the later was transferred into cell nuclei to promote the expression of cytokines [18]. Our results also showed that transcription level of pro-inflammatory factors IL-1β and IL-6 was significantly increased by NF-κB activation induced by rTsgal, which might lead to intestinal inflammation and mucosal barrier dysfunction, consequently accelerated larval penetration of gut mucosa [44, 55]. It has been reported that pro-inflammatory factors can degrade tight junction proteins of gut epithelium after the onset of inflammation, allowing further disruption of intestinal barrier [61]. The upregulation of pro-inflammatory factor secretion after activation of TLR-4-ERK1/2-NF-κB pathway might also destroy tight junction proteins to damage the intestinal mucosal barrier, thus facilitated the *T. spiralis* invasion of gut mucosa.

To further confirm that the binding of rTsgal to TLR-4 activated the MAPK-NF-κB pathway in Caco-2 cells, TLR-4 receptor specific inhibitor TAK-242 and NF-κB specific inhibitor PDTC were used to ascertain whether the activation of TLR-4-ERK1/2-NF-κB pathway could be inhibited by the two inhibitors. TAK-242 binds directly to the intracellular structural domain of TLR-4, and the binding site was located in the intracellular cysteine, preventing the binding of TLR-4 to downstream molecules, thereby inhibiting the downstream cascade reaction [45, 62]. Our results indicated that TAK-242 obviously abrogated the rTsgal-induced activation of NF-κB/MAPK pathway in Caco-2 cells, as demonstrated that both 20 µM and 25 µM TAK-242 inhibited rTsgal-induced up-regulation of p-ERK1/2, p-NF-κB p65 and NF-κB p65. It has been shown that post-translational modifications of NF-κB are important for playing transcriptional activity, especially phosphorylation of the p65 subunit [63], so PDTC was also chosen as an inhibitor of NF-κB pathway. PDTC can prevent NF-κB activation and transcription of downstream cytokines by inhibiting the dissociation of IκB with RelA (p65). Several studies have showed that PDTC inhibited NF-κB pathway in Caco-2 cells at concentrations of 100 to 500 mM, and 300 mM PDTC determined by CCK-8 assay was used in this study.

The Caco-2 cells were pre-incubated with 25 µM TAK-242 and 300 mM PDTC for 2 h, then the cells were incubated with rTsgal for 48 h. The transcription and expression levels of TLR-4 in Caco-2 cells were significantly down-regulated compared to their solvent (DMSO or PBS) control group, indicating that TAK-242 and PDTC could inhibit rTsgal to activate TLR-4. To further observe the suppressive effect of the two inhibitors on the rTsgal-activated up-regulation of MAPK-NF-κB pathway in Caco-2 cells, Caco-2 cells were incubated with rTsgal for 0.5 h after they were pretreated by inhibitors, and the changes of phosphorylated levels of p65 and ERK1/2 were also observed. The results showed that both TAK-242 and PDTC distinctly inhibited the phosphorylation of the two proteins. After suppressing activation of MAPK-NF-κB pathway through inhibiting TLR-4, TAK-242 also inhibited the phosphorylation of NF-κB p65 and the entry of NF-κB into the nucleus, which ultimately affected the transcription levels of downstream pro-inflammatory cytokines (IL-1β and IL-6). This phenomenon was also present in intestinal inflammation resulted from intestinal flora. When Caco-2 cells were pretreated with TAK-242, the effect of intestinal flora indole derivatives (IAA) on cellular inflammatory stimulation was significantly inhibited, leading to a significant down-regulation of the IAA-induced pro-inflammatory factor TNF-α [46]. In macrophages, TAK-242 inhibited the LPS-triggered TLR-4-ERK1/2-NF-κB signaling pathway and reduced the production of pro-inflammatory mediators such as IL-6, TNF-α, IL-1β, ROS, NO and PGE2 [64]. Although PDTC is an inhibitor of NF-κB, but the inhibition of NF-κB also regulates ERK1/2 activation, for example, in human bronchial epithelial cells, PDTC not only down-regulated the activation of ERK1/2 by LPS, NF-κB p65 was also inhibited [65]. This phenomenon may be due to the fact that NF-κB affects the expression of cytokines as well as various immunomodulatory molecules such as TLR-4, which indirectly affects the phosphorylation of ERK1/2. Therefore, PDTC and TAK-242 inhibited the Tsgal-induced secretion of pro- and anti-inflammatory factors, thereby attenuated the intestinal inflammation.

Our results also showed that rTsgal promoted inflammatory response of Caco-2 cells after binding to TLR-4 and activating the MAPK-NF-κB pathway, up-regulating transcription levels of pro-inflammatory factor (IL-1β and IL-6) and down-regulating transcription levels of anti-inflammatory factor (TGF-β), and this process could be inhibited by TAK-242 and PDTC. Therefore, the effect of TLR-4-MAPK-NF-κB pathway on *Trichinella* invasion of gut epithelium was investigated in this study. An in vitro larval invasion of Caco-2 monolayer was performed. After the cell monolayer was treated with TAK-242 and PDTC, respectively, the IIL were added to semi-solid medium and co-cultured with the monolayer. The results showed that both inhibitors not only inhibited the IIL invasion of cell monolayer, but also abrogated the role of rTsgal promoting IIL invasion.

Animal challenge results showed that when the mice were pretreated with TAK-242 and PDTC, and then challenged with *T. spiralis*, the two inhibitors significantly inhibited the mRNA expression level of TLR-4 and NF-κB p65, reduced production of pro-inflammatory cytokines (IL-1β and IL-6) and increased secretion of anti-inflammatory cytokine (TGF-β) in infected mice. Moreover, the two inhibitors obviously alleviated intestinal inflammation and decreased the number of Paneth cells in gut epithelium; the intestinal AW burden was evidently reduced. Paneth cells are located at the base of enteral crypt, and secretory granules in cytoplasm prevent the worm penetration into the crypt [66]. Paneth cells are the main component of intestinal innate immunity; their secreted granules contain different antimicrobial materials (for example, defensin, lysozyme and sIgA), they are also significant component of inflammatory reaction. Therefore, Paneth cells act a significant function to maintaining the integrity of gut mucosal barrier [67, 68]. The results suggested that the small molecule inhibitors (TAK-242 and PDTC) could be considered as a therapeutic adjuvant to reduce intestinal inflammation at early stage of *Trichinella* infection. The results further demonstrated that Tsgal binding to TLR-4 in gut epithelium induced intestinal inflammatory reaction, and mediated larval invasion of gut mucosa via activating MAPK-NF-κB pathway. Therefore, Tsgal might be regarded as a candidate molecular target of vaccine for blocking *T. spiralis* invasion and infection.

In conclusion, specific binding between rTsgal and TLR-4 in Caco-2 cells was verified by IIF, GST pull-down and Co-IP assay, indicating that TLR-4 in Caco-2 cells is a Tsgal binding partner. The results of qPCR and Western blotting showed that binding of rTsgal and TLR-4 up-regulated the TLR-4 expression level in Caco-2 cells, activated p-NF-κB p65 and p-ERK1/2. The activation of inflammatory signaling pathway TLR-4-MAPK-NF-κB by rTsgal resulted in obvious up-regulation of pro-inflammatory cytokines (IL-1β and IL-6) and down-regulation of anti-inflammatory cytokine TGF-β in Caco-2 cells, and induced intestinal mucosal inflammation. TLR-4 specific inhibitor TAK-242 and NF-κB specific inhibitor PDTC significantly inhibited the activation of TLR-4 receptor and MAPK-NF-κB pathway. Moreover, the two inhibitors also inhibited the expression of IL-1β and IL-6, and increased TGF-β. TAK-242 and PDTC also obviously inhibited larval invasion of cell monolayer and gut mucosa, abrogated the role of rTsgal promoting larval invasion, and alleviated intestinal inflammation. The results showed that Tsgal binding to TLR-4 in gut epithelium activated MAPK-NF-κB pathway, induced the expression of TLR-4 and pro-inflammatory cytokines, induced intestinal inflammation and mediated larval invasion of gut mucosa. Tsgal might be regarded as a candidate molecular target of vaccine against *T. spiralis* enteral invasive stage.

### Supplementary Information

**Additional file 1. Cell viability assessed by CCK-8 kit.** The rTsgal (0, 10, 20, 30, 40 and 50 μg/mL) and IIL crude antigens (0, 10, 20, 30 and 40 μg/mL) were incubated with Caco-2 cells for 24 and 48 h to assess the cell viability. The absorbance (OD value) at 450 nm was regarded as the cell proliferation index. The data were from three independent experiments, and are presented as the mean ± standard deviation (SD).

Additional file 2. The viability of Caco-2 cells treated by different concentrations of TAK-242 (A) and PDTC (B) *P< 0.01 compared to the PBS group.

**Additional file 3. Intestinal pathological changes at 7 days after** ***T. spiralis*** **infection by HE staining.** Intestinal sections were stained by haematoxylin and eosin (HE) and examined under microscopy. Enteral section from the solvent (DMSO or PBS) control group exhibited the destruction of villous structure, villous edema, and inflammatory cell infiltration in the villus, and increased number of Paneth cells and intracellular particles. Intestinal tissue of two inhibitor groups revealed the relative normal villous structure, as demonstrated narrower enteral villus width and less Paneth cells (blue arrows). Scale bars = 200 μm.

**Additional file 4. Intestinal pathological changes at 6 days in mice pretreated with TAK-242 (A, B) and PDTC (C, D) after** ***T. spiralis*** **infection.** **A**,** C** Number of intestinal Paneth cells;**B**,** D** Width of enteral villi. *P < 0.05 relative to the solvent (DMSO or PBS) control groups.

## Abbreviations

AEC: an enhanced chemiluminescent kit
AW: adult worm
BSA: bovine serum albumin
Co-IP: co-immunoprecipitation
CRD: carbohydrate recognition domain
CTL: CAg:crude antigens
DAB: 3,3′-diaminobenzidine tetrahydrochloride
DAPI: 4′,6-diamidino-2-phenylindole
DMEM: Dulbecco’s modified eagle medium
DMSO: dimethyl sulfoxide
ESP: excretory/secretory products
dpi: days post-infection
FBS: fetal bovine serum
hpi: hours post-infection
HRP: horseradish peroxidase
GAPDH: glyceraldehyde 3-phosphate dehydrogenase
GSH: glutathione
GST: glutathione mercaptotransferase
IBD: inflammatory bowel disease
IEC: intestinal epithelium cells
IIF: indirect immunofluorescenc
IIL: intestine infectious larvae
ML: muscle larvae
MW: molecular weight
NBL: newborn larvae
NC: nitrocellulose
PAMP: pathogen-associated pattern recognition molecule
PBMC: peripheral blood mononuclear cells
PDTC: pyrrolidinecarbodithioic acid
PRRs: pattern recognition receptors
qPCR: real-time quantitative PCR
SD: standard deviation
TBST: Tris-buffered saline containing 0.05% Tween
TLRs: Toll-like receptors
Tsgal: *T. spiralis* galectin
