# Supplementary material for: Trichinella spiralis galectin binding to toll-like receptor 4 induces intestinal inflammation and mediates larval invasion of gut mucosa
Source: Vet Res. 2023 Nov 27;54:113. doi: 10.1186/s13567-023-01246-x (PMC10680189; doi:10.1186/s13567-023-01246-x)
Supplement: Supplementary file 1 — Additional file 1. Cell viability assessed by CCK-8 kit. The rTsgal (0, 10, 20, 30, 40 and 50 μg/mL) and IIL crude antigens (0, 10, 20, 30 and 40 μg/mL) were incubated with Caco-2 cells for 24 and 48 h to assess the cell viability. The absorbance (OD value) at 450 nm was regarded as the cell proliferation index. The data were from three independent experiments, and are presented as the mean ± standard deviation (SD). [file 13567_2023_1246_MOESM1_ESM.docx]

**
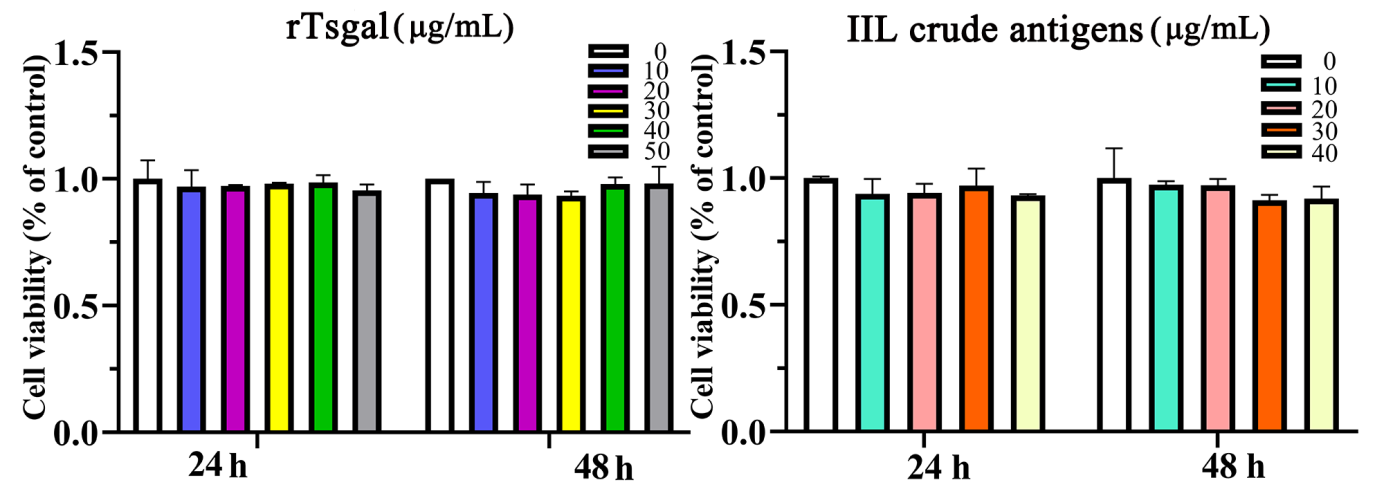
**

**Additional file 1. Cell viability assessed by CCK-8 kit**. The rTsgal (0, 10, 20, 30, 40 and 50 μg/mL) and IIL crude antigens (0, 10, 20, 30 and 40 μg/mL) were incubated with Caco-2 cells for 24 and 48 h to assess the cell viability. The absorbance (OD value) at 450 nm was regarded as the cell proliferation index. The data were from three independent experiments, and are presented as the mean ±standard deviation (SD).
