# Supplementary material for: Trichinella spiralis galectin binding to toll-like receptor 4 induces intestinal inflammation and mediates larval invasion of gut mucosa
Source: Vet Res. 2023 Nov 27;54:113. doi: 10.1186/s13567-023-01246-x (PMC10680189; doi:10.1186/s13567-023-01246-x)
Supplement: Supplementary file 2 — Additional file 2. The viability of Caco-2 cells treated by different concentrations of TAK-242 (A) and PDTC (B) *P< 0.01 compared to the PBS group. [file 13567_2023_1246_MOESM2_ESM.docx]

**
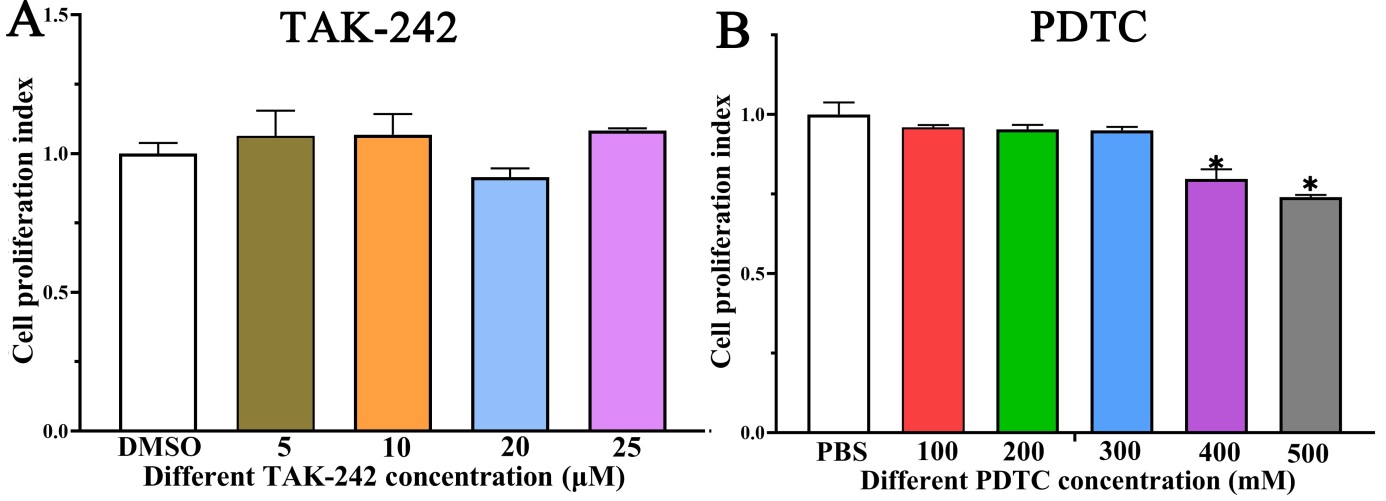
**

**Additional file 2. The viability of Caco-2 cells treated by different concentrations of TAK-242 (A) and PDTC (B)** **P* < 0.01 compared to the PBS group.
