# Supplementary material for: Trichinella spiralis galectin binding to toll-like receptor 4 induces intestinal inflammation and mediates larval invasion of gut mucosa
Source: Vet Res. 2023 Nov 27;54:113. doi: 10.1186/s13567-023-01246-x (PMC10680189; doi:10.1186/s13567-023-01246-x)
Supplement: Supplementary file 3 — Additional file 3. Intestinal pathological changes at 7 days after T. spiralis infection by HE staining. Intestinal sections were stained by haematoxylin and eosin (HE) and examined under microscopy. Enteral section from the solvent (DMSO or PBS) control group exhibited the destruction of villous structure, villous edema, and inflammatory cell infiltration in the villus, and increased number of Paneth cells and intracellular particles. Intestinal tissue of two inhibitor groups revealed the relative normal villous structure, as demonstrated narrower enteral villus width and less Paneth cells (blue arrows). Scale bars = 200 μm. [file 13567_2023_1246_MOESM3_ESM.docx]

**
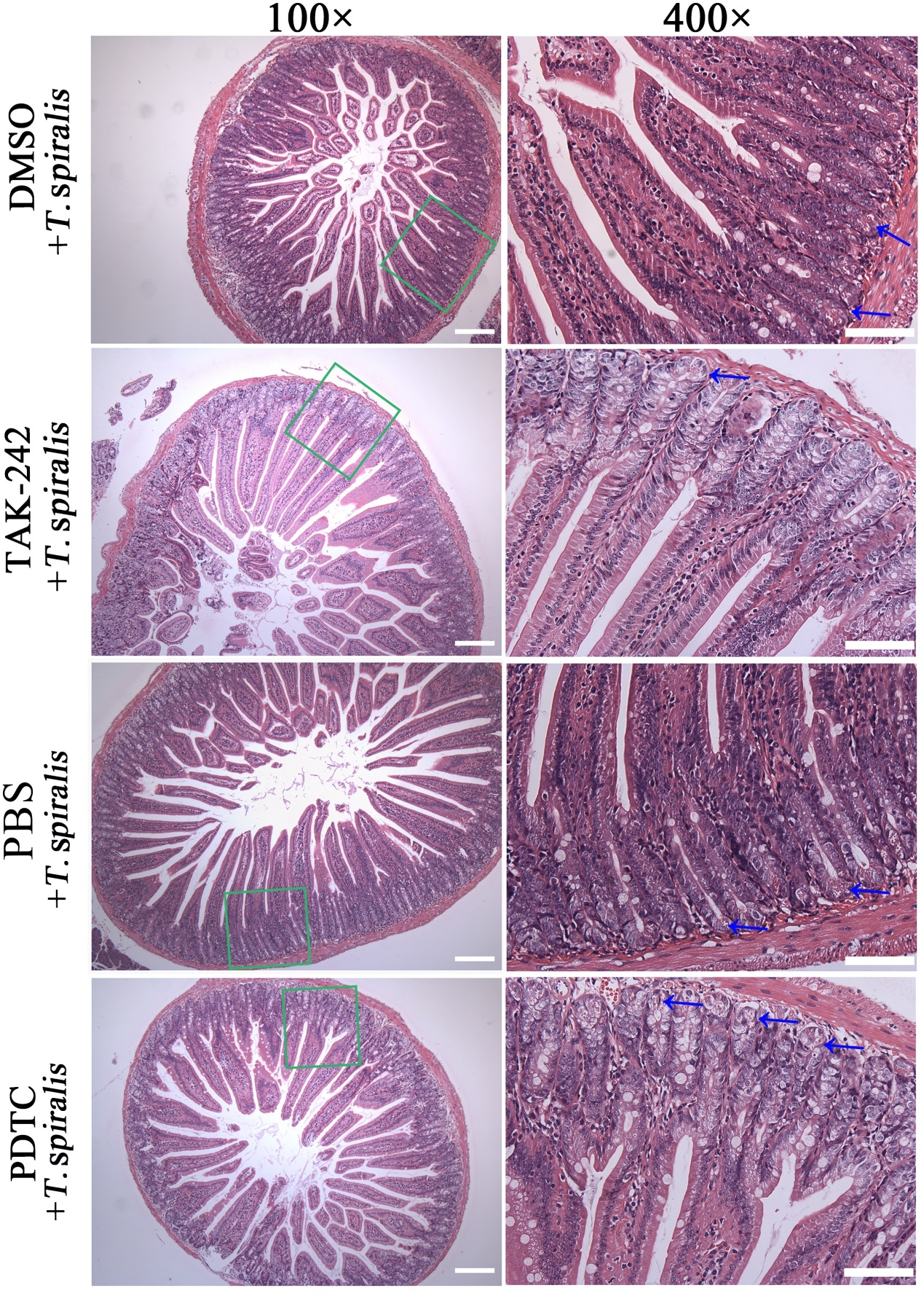
**

**Additional file 3. Intestinal pathological changes at 7 days after *T. spiralis* infection by HE staining.** Intestinal sections were stained by haematoxylin and eosin (HE) and examined under microscopy. Enteral section from the solvent (DMSO or PBS) control group exhibited the destruction of villus structure, villous edema, and inflammatory cell infiltration in the villus, and increased number of Paneth cells and intracellular particles. Intestinal tissue of two inhibitor groups revealed that narrower enteral villus width and less Paneth cells (blue arrows). Scale bars = 200 μm.
