# Supplementary material for: Trichinella spiralis galectin binding to toll-like receptor 4 induces intestinal inflammation and mediates larval invasion of gut mucosa
Source: Vet Res. 2023 Nov 27;54:113. doi: 10.1186/s13567-023-01246-x (PMC10680189; doi:10.1186/s13567-023-01246-x)
Supplement: Supplementary file 4 — Additional file 4. Intestinal pathological changes at 6 days in mice pretreated with TAK-242 (A, B) and PDTC (C, D) after T. spiralis infection. A, C Number of intestinal Paneth cells;B, D Width of enteral villi. *P < 0.05 relative to the solvent (DMSO or PBS) control groups. [file 13567_2023_1246_MOESM4_ESM.docx]

**
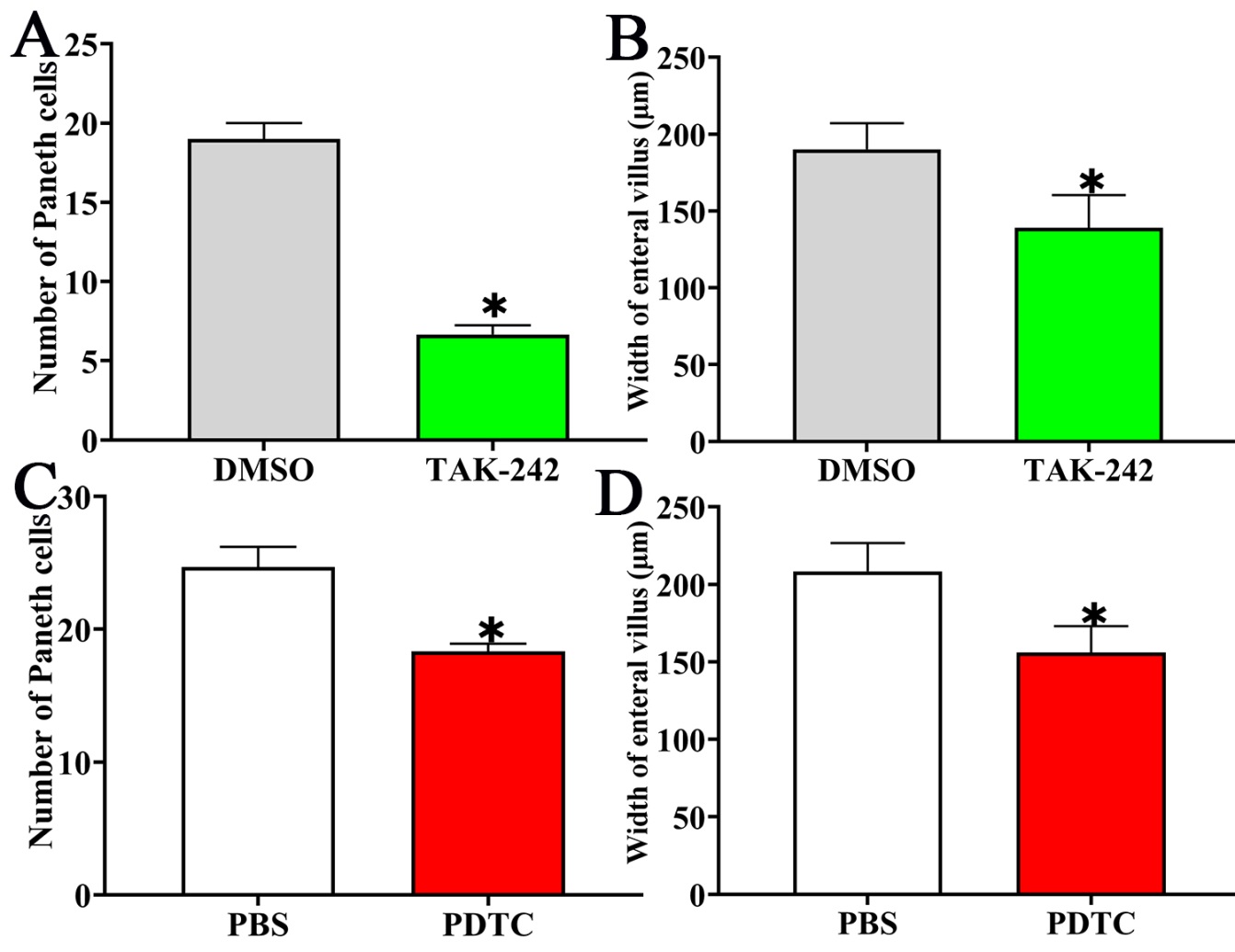
**

**Additional file 4. Intestinal pathological changes at 6 days in mice pretreated with TAK-242 (A, B) and PDTC (C, D) after *T. spiralis* infection. A** and **C:** Number of intestinal Paneth cells; **B** and **D:** Width of enteral villi. **P* < 0.05 relative to the solvent (DMSO or PBS) control groups.
